# Reconstruction of Three-Dimensional Conformations of Bacterial ClpB from High-Speed Atomic-Force-Microscopy Images

**DOI:** 10.3389/fmolb.2021.704274

**Published:** 2021-08-05

**Authors:** Bhaskar Dasgupta, Osamu Miyashita, Takayuki Uchihashi, Florence Tama

**Affiliations:** ^1^ Computational Structural Biology Research Team, RIKEN-Center for Computational Science, Kobe, Japan; ^2^ Institute for Glyco-core Research (iGCORE), Nagoya University, Nagoya, Japan; ^3^ Exploratory Research Center on Life and Living Systems (ExCELLS), National Institutes of Natural Sciences, Okazaki, Japan; ^4^ Department of Physics, Graduate School of Science, Nagoya University, Nagoya, Japan; ^5^ Institute of Transformative Bio-Molecules, Nagoya University, Nagoya, Japan

**Keywords:** ClpB, 3D modeling, Monte-Carlo sampling, Gaussian mixture model, atomic-force-microscopy image analysis

## Abstract

ClpB belongs to the cellular disaggretase machinery involved in rescuing misfolded or aggregated proteins during heat or other cellular shocks. The function of this protein relies on the interconversion between different conformations in its native condition. A recent high-speed-atomic-force-microscopy (HS-AFM) experiment on ClpB from *Thermus thermophilus* shows four predominant conformational classes, namely, open, closed, spiral, and half-spiral. Analyses of AFM images provide only partial structural information regarding the molecular surface, and thus computational modeling of three-dimensional (3D) structures of these conformations should help interpret dynamical events related to ClpB functions. In this study, we reconstruct 3D models of ClpB from HS-AFM images in different conformational classes. We have applied our recently developed computational method based on a low-resolution representation of 3D structure using a Gaussian mixture model, combined with a Monte-Carlo sampling algorithm to optimize the agreement with target AFM images. After conformational sampling, we obtained models that reflect conformational variety embedded within the AFM images. From these reconstructed 3D models, we described, in terms of relative domain arrangement, the different types of ClpB oligomeric conformations observed by HS-AFM experiments. In particular, we highlighted the slippage of the monomeric components around the seam. This study demonstrates that such details of information, necessary for annotating the different conformational states involved in the ClpB function, can be obtained by combining HS-AFM images, even with limited resolution, and computational modeling.

## Introduction

For a healthy cell, specific machinery relieves the effect of stress and disease on the cell. One such biomolecular machine that helps to recover cells from the deposition of aggregated proteins due to heat and proteotoxic stresses is the Hsp100 chaperon in cooperation with Hsp70 (Glover and Lindquist, 1998; Haslberger et al., 2010; Doyle et al., 2013; Mogk et al., 2018). The Hsp100 proteins are prevalent in bacteria (known as ClpB), or Yeast (known as Hsp104), and belongs to the AAA+ superfamily of ATPase proteins, hosting two ATPase domains per monomer in its hexameric structure (Deville et al., 2017; Deville et al., 2019). The disaggregation function of Hsp100 proteins takes place when the substrate proteins pass through its central pore which involves large-scale conformational changes of Hsp100 (Weibezahn et al., 2004; Gates et al., 2017; Rizo et al., 2019). Although the mechanism of such conformational changes has gained recent attention, the characterization of the dynamics including many underlying conformational states is still limited (Uchihashi et al., 2018).

In *E. coli* ClpB and Yeast Hsp104, in addition to the two ATPase domains (AAA1+ and AAA2+), each monomer includes an N-terminal domain associated with substrate binding (Barnett et al., 2005; Lee et al., 2007; Mizuno et al., 2012) and a long coiled-coil domain—acting as a “propeller” to bind Hsp70 (Carroni et al., 2014; Mogk et al., 2015). The AAA1+ and AAA2+ domains constitute the hexameric core structure with a pore in the middle, which has been shown to bind a casein substrate from cryo-electron microscopy (cryo-EM) reconstruction (Gates et al., 2017; Rizo et al., 2019). The AAA1+ and AAA2+ domains incorporate Walker A and B motifs that are responsible for ATP binding and hydrolysis, and cooperative ATP binding is associated with the structural changes in the hexamer (Mogk et al., 2003). The structural studies of ClpB/Hsp104 revealed its hexameric form, however, high-speed-atomic-force-microscopy (HS-AFM) imaging also indicates that the hexamer is fragile and breaks frequently as required in the disaggregation mechanism (Uchihashi et al., 2018). The non-rigid nature of the hexamer is also observed in a recent cryo-EM analysis revealing a spiral two-tier AAA+ ring of interaction (Yokom et al., 2016).

In the HS-AFM experiments, the structural dynamics of ClpB from *Thermus thermophilus* was investigated (Uchihashi et al., 2018). The HS-AFM images clearly indicated that the hexamer ring is fragile to form not only the round closed structure but also open or spiral conformations. The HS-AFM images include four main conformational classes, *open*, *closed*, *spiral,* and *half-spiral*. In the closed or spiral structure, a common feature is a seam between two monomers, along which the monomers separate to form the open conformations. The half-spiral architecture resembles a dimer of trimer, forming an additional seam in the opposite end. However, it should be noted that such conformational classes were inferred from the HS-AFM images, in which only a partial structure of ClpB viewed from the top was observed. Therefore, we aim to model the three-dimensional ClpB structures to visualize and interpret the salient feature of the hexameric structures and further help relate the structure of ClpB to its function.

Hybrid modeling approaches, combining computation and experiment have been developed to generate 3D models from low-resolution data (Rout and Sali, 2019; Srivastava et al., 2020). In such approaches, data from multiple sources are combined through the lens of computational sampling, aiming to better interpret experimental data. Even for low-resolution structural data, the usage of computational modeling enables us to discuss the function of biomolecules in terms of 3D models. Such hybrid or integrative modeling techniques have been widely used, where they are applied to recover structural details from small-angle X-ray scattering profile (Gorba and Tama, 2010; Derevyanko and Grudinin, 2014; Schindler et al., 2016; Ekimoto and Ikeguchi, 2018; Chen et al., 2019), cross-linking mass spectrometry (Faini et al., 2016; Degiacomi et al., 2017), cryo-EM (Trabuco et al., 2008; Grubisic et al., 2010; Miyashita et al., 2017; Kim et al., 2019; Malhotra et al., 2019), X-ray free-electron laser (XFEL) (Tokuhisa et al., 2016; Nagai et al., 2018; Nakano et al., 2018) and AFM (Amyot and Flechsig, 2020; Dasgupta et al., 2020; Niina et al., 2020; Fuchigami et al., 2021) studies. Some of these methods aim to recover structural details from experimental data by simulating conformational changes from a known conformational state. Recently, we have developed such an approach to relate 3D conformational changes embedded in theoretical AFM images (Dasgupta et al., 2020). In this study, we apply our algorithm to experimental ClpB AFM images.

Our algorithm uses Monte-Carlo (MC) sampling to fit an initial low-resolution 3D structural model to a target AFM image. Structural models are represented at low-resolution using 3D Gaussian density distributions since the target AFM data is a low-resolution image usually from a large system. A 3D Gaussian mixture model (3D-GMM), derived from an atomically detailed structure, is used as the initial low-resolution model (Kawabata, 2008). It should be noted that an atomic structure is not necessary to generate the initial 3D-GMM. The MC sampling algorithm is based on three crucial factors. First, during the optimization, we need to generate a pseudo-AFM image from our 3D models. 3D-GMM can be used to rasterize over the set of kernels generating a low-resolution pseudo-AFM image. Second, we need to compare this generated pseudo-AFM image to the target AFM image of a given protein. Third, candidate models generated during the Monte Carlo sampling need to be evaluated to keep the model structurally compact.

**FIGURE 1:**
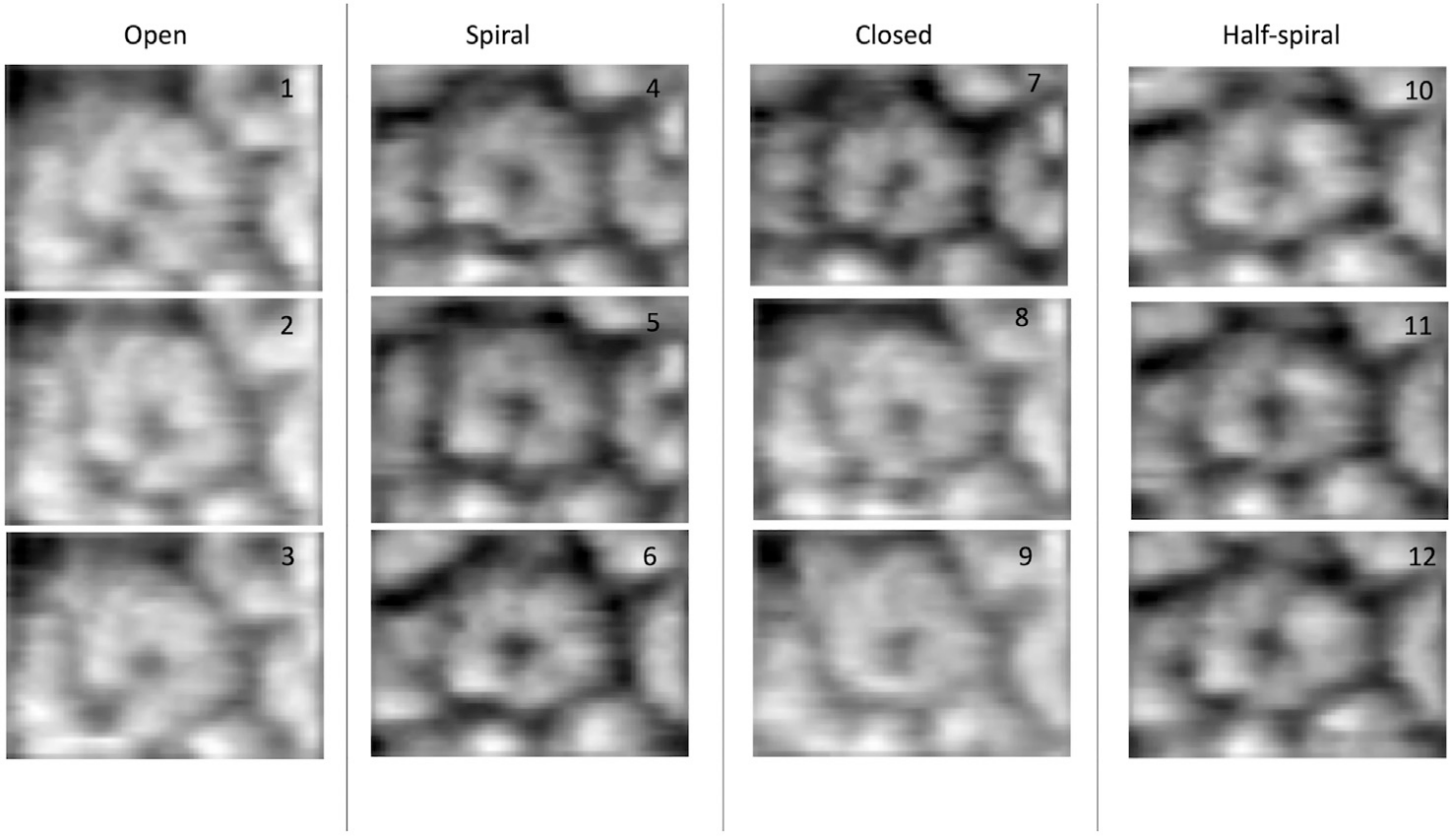
Experimental AFM images used for 3D structure modeling (Uchihashi et al., 2018).

The HS-AFM experiments on ClpB dynamics were performed under near-physiological conditions revealing a variety of ClpB conformations, which were significantly different from either of the known conformations (Uchihashi et al., 2018). In our current study, we started from two atomically detailed conformations obtained from cryo-EM experiments, an asymmetric non-rigid two-tier spiral structure of Hsp104 from Yeast (Yokom et al., 2016) and a symmetric closed ring conformation of ClpB from *E. coli* (Deville et al., 2017). We modified our algorithm to consider both conformations as initial models and perform sampling based on a mechanical potential defined by combining the initial spiral and closed ring conformations. We performed 3D modeling on four different conformational classes observed in the HS-AFM experiments (Figure 1, Supplementary Table 1). The reconstructed 3D models from our sampling can be used to detect salient features within ClpB conformations. Moreover, we decoded some finer details of the ClpB hexameric architecture, which cannot be clearly observed from HS-AFM images. Lastly, we could also interpolate between different conformational classes to compare novel ClpB structures. These results demonstrate that our 3D structure modeling approach from AFM images can be applied to experimental data, providing a new approach to study conformational transitions in macromolecular complexes through AFM-computation hybrid modeling.

## Materials and Methods

### Preparation of Initial Models

**FIGURE 2:**
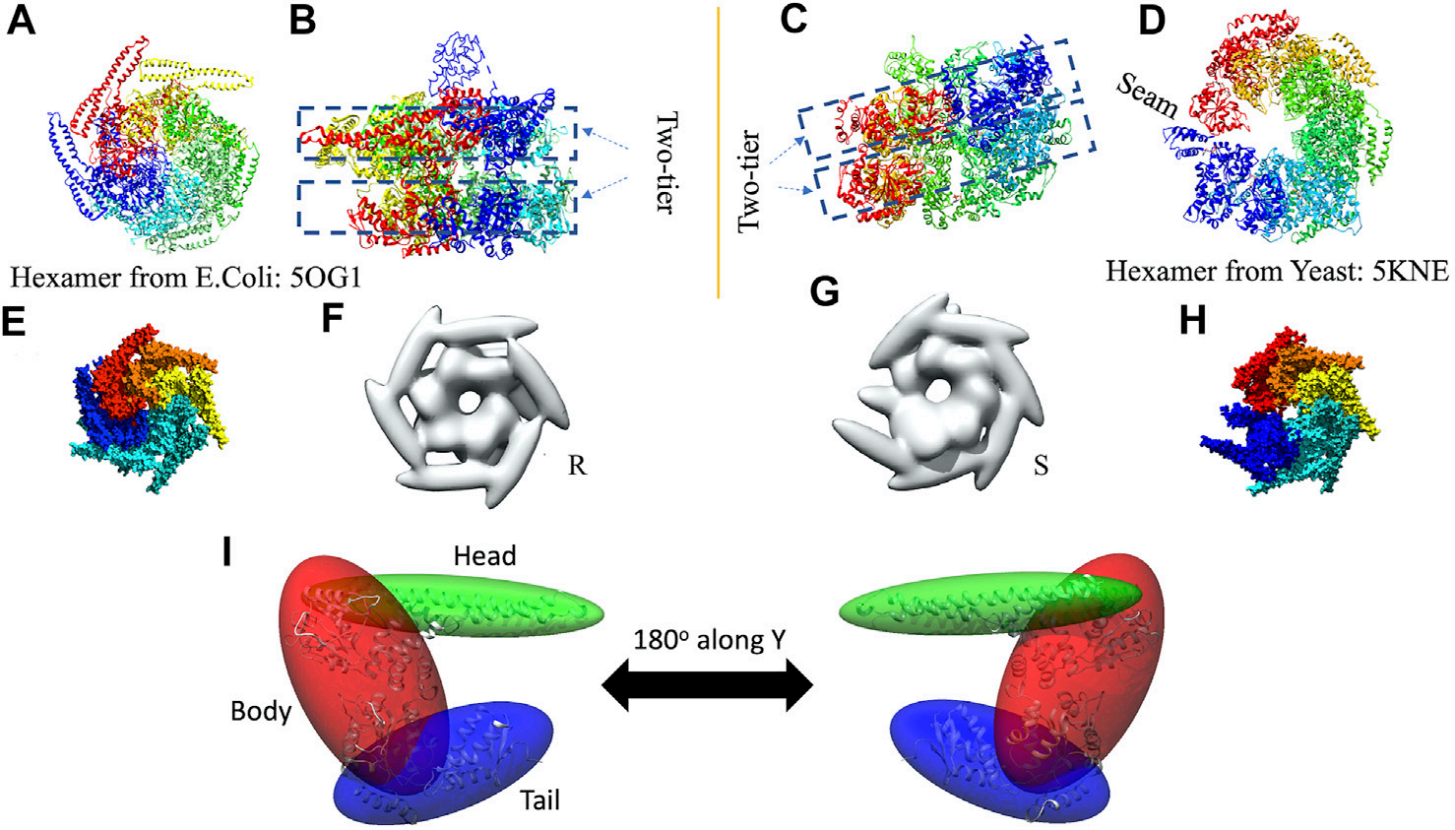
Hexameric structures selected as initial models from *E. coli* **(A,B)** and Yeast **(C,D)** from 5OG1 and 5KNE, respectively. In **(A)** and **(D)** the structures are shown from the top and in **(B,C)**, a side-view is shown describing their two-tier structure. In **(D)** a seam is shown between two neighboring chains denoted by chains “A” (in blue) and “F” (in red). The corresponding seam is less prominent in 5OG1 (see Figure 2A). In the middle row **(E–H)**, the models with *T. thermophilus* monomer (from 1QVR, chain A) are shown, and in **(I)** the 3-kernel model for 1QVR, chain A is shown (with the atomic structure embedded); the head, body, and tail kernels are annotated and shown in different colors. In **(E,H)**, the atomic hexamer models after superimposition to 5OG1 and 5KNE are shown from a top view, respectively. In **(F,G)**, the 18-kernel representations based on 5OG1 and 5KNE which are referred to as R and S initial models in the text.

In 3D modeling against the AFM images, we have used two initial models based on structures originally obtained from cryo-EM reconstructions of Yeast ClpB, which adopts a two-tier spiral conformation (PDB ID: 5KNE) (Yokom et al., 2016) and *E. coli* ClpB, whose conformation is a symmetric closed ring (PDB ID: 5OG1) (Figures 2A–D) (Deville et al., 2017).

These two structures are sequentially different from the *T. thermophilus* construct used in the HS-AFM experiment (Uchihashi et al., 2018). To mimic the experimental construct, the missing residues in 1QVR (a trimeric structure of ClpB from *T. thermophilus* with sequence identity to chain A of 5KNE is 44.8% and to chain A of 5OG1 is 56.1%), chain A were modeled with Modeller using the automodel class (residues 1 to 3, 851 to 854 in the terminals and three loop regions from residues 235 to 245, 272 to 290, and 637 to 650) (Supplementary Figure 1) (Martí-Renom et al., 2000; Webb and Sali, 2014). To prepare the initial structures based on 5KNE and 5OG1, we first superposed 1QVR chain A to each of the chains of 5KNE and 5OG1 (chains A to F) by using Chimera matchmaker (see Supplementary Section 2A, Supplementary Table 2) (Pettersen et al., 2004; Meng et al., 2006). This is followed by deletion of the N-terminal regions (residue 1–165) generating two hexameric ClpB arrangements, i.e., a closed ring and a spiral conformation. Finally, the whole complex was oriented so that the C-terminal regions face towards the bottom as observed in the HS-AFM experiments (Uchihashi et al., 2018) (Figures 2E,H).

The AFM images are of nanometer resolution, therefore conformational transitions cannot be discussed with atomic-level details and thus we employ a coarse-grained three-dimensional Gaussian mixture model. In this technique a polypeptide chain is described by a weighted sum of three-dimensional Gaussian kernels. A Gaussian kernel is parameterized by its center and a covariance matrix. The volume of the kernel within a certain threshold is geometrically represented by an ellipsoid (see Supplementary Section 2B). The parameters of the mixture model can be obtained by expectation-maximization optimizing algorithm. In the current study, we used “gmconvert” software (Kawabata, 2008; Kawabata, 2018) to obtain such Gaussian mixture models.

We employ a Gaussian mixture model defined by an 18 Gaussian kernel arrangement, where a chain in the hexamer is described by 3 kernels (Supplementary Figure 1, Figure 2I). Each of the domains of the ClpB monomer is included in different Gaussian kernels. The coiled-coil domain of ClpB is included in a long elliptical kernel which we refer to *head* kernel. The C-terminal domain of ClpB is included in the *tail* kernel. The nucleotide-binding domains AAA1+ and AAA2+ together with the linker domain are included in the largest kernel that we refer to as *body* kernel. Looking from the top, as imaged in the HS-AFM experiment, the upper part of the body kernel hosts the AAA1+ domain while the bottom part hosts the AAA2+ domain. The coiled-coil domain is found at the top and the C-terminal region at the bottom. These 18-kernel Gaussian mixture models starting from 5OG1 and 5KNE are used as initial conformations to model AFM images and are hereafter are referred to as R (closed symmetric Ring conformation) and S (Spiral asymmetric conformation), respectively (Figures 2F,G). We call each of the six chains in the models A to F, where F and A are across the seam.

In the closed ring or spiral conformation, a common feature is a seam between two monomers. Therefore, we manually rotated around the *z*-axis of the S model, for which the seam is more prominent, to align it with the seam observed in the AFM images (see Supplementary Sections 2C,D, Supplementary Tables 1, 3). For the R model, we applied a z-rotation identical to that performed on the S model. Such rotated models were used to start the MC sampling. This protocol works well (in terms of final converged models) for the AFM images with the annotations spiral, open and close, however, poor convergence was observed for half-spiral cases. In such a case, the R model also needed to be rotated, independently of the S model. The z-rotations applied to different models are given in Supplementary Table 3.

### Kernel Position Restraints for Sampling

In the MC algorithm, a random move is applied to one of the kernels and the restraint score for this new model is calculated to determine whether the model should be kept. This score is based on an empirical scoring function to model attraction and repulsion between kernels to ensure that the kernels would not overlap or move too far apart. The restraint scores are defined for each of the kernel pairs using their overlap values. Each term has the lowest score when the overlap value is the average of those for the two initial models (see Supplementary Section 4). The restraint score is given by an asymmetric harmonic function, with different curvature parameters for attraction and repulsion. These parameters are selected based on the types of kernel pairs. The restraints between the three kernels within each chain are set to be strongest (see Figure 2I for the kernel definitions) so that three domains keep the overall shape of the monomer while maintaining enough flexibility to allow conformational transitions. The interchain restrains between the neighboring “body” kernels are set to be less restrictive than intrachain pairs. The tail-kernels repulsion parameters between neighboring chains are set to be identical to the one between neighboring body kernels since the “tail” kernels closely follow “body” kernels. The “head” kernels comprising coiled-coil domain are least restricted owing to their flexible nature. One important aspect of the parameters is how we define the interaction between the chains A and F, which completes the circular arrangement. AFM results, as well as previous atomic structures of ClpB, revealed the possible presence of a seam between these chains. In addition, for the open-class conformation described in AFM images, those two chains are separated. Therefore, we assume that the interaction between the chains A and F is special in that the attraction is weaker. The details of those parameters are given in Supplementary Section 4, Supplementary Table 4.

Apart from the above attraction-repulsion scoring, to incorporate the connectivity of the underlying molecular structure into modeling, we also ensure that the intersections between kernels in the initial model remained. Such intersections can be viewed as hinges between kernels. To do so, a pair of phantom particles is assigned to the intersection site, one from each of the overlapping kernels (Supplementary Figure 4). Those phantom particles are initially on top of each other, but due to sampling, they may go far and thereby breaks the connection. We ensure, via a connectivity restraint, that the distance between two such phantom particles should be within 5 Å and each of them always falls within the overlapping region.

### Sampling against AFM Image

**FIGURE 3:**
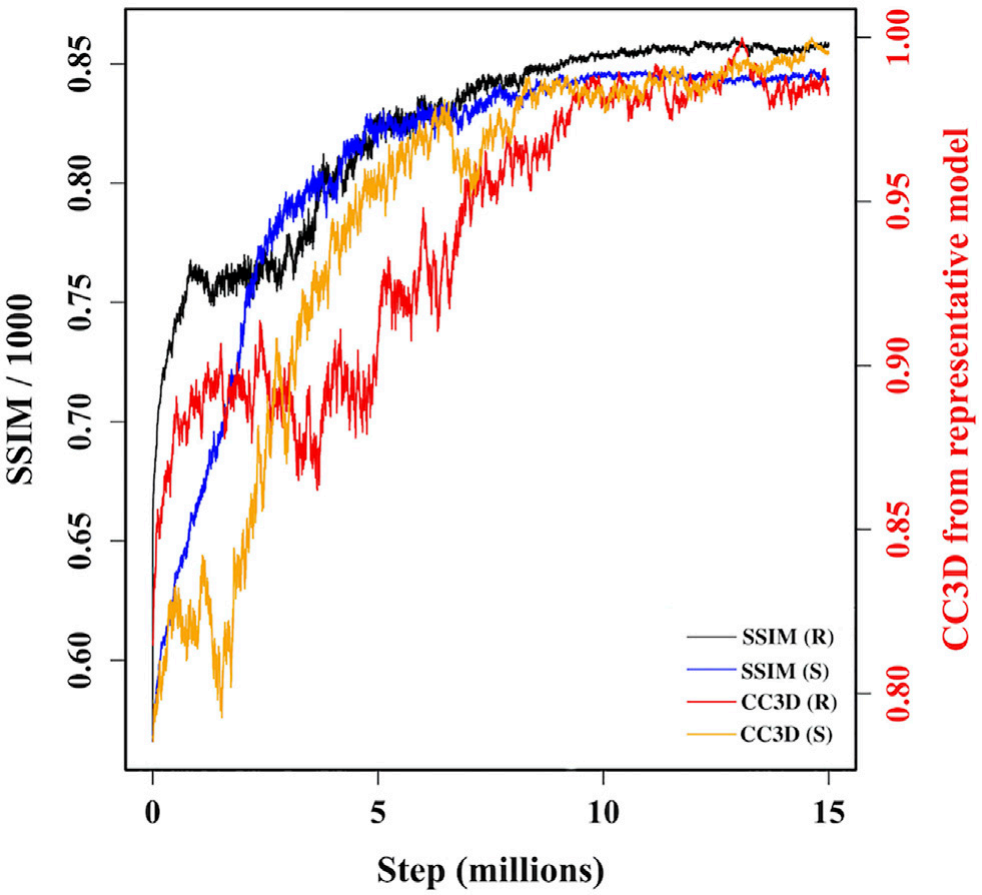
A plot of variation of similarities between candidate models along two MC trajectories, initiated from R and S models, and target AFM image 7. The left vertical axis shows variation of SSIM between candidate models and target AFM image. The right vertical axis shows variation of the correlation coefficient after rigid-body fitting of kernel centers between intermediate candidate models and final converged model.

During MC sampling we compare a pseudo-AFM image from the current candidate model (aka candidate image) to the target AFM input image. In this study, we used a more robust image similarity measure—the structural similarity metric (SSIM) (Wang et al., 2004; Wang and Bovik, 2009) instead of L1-norm in our previous study (Dasgupta et al., 2020). The SSIM takes its maximum value of 1.0 when two images are identical. The SSIM between the candidate image and target AFM image is first calculated. Then the candidate model (in 3D) is updated by applying a random move to one of the 18 Gaussian kernels. After applying such a move, we either accept or reject the updated model based on the change in the restraint score. The change in the score is converted to a probability associated with a temperature parameter. If the updated model is accepted in this first step, then we measure the similarity between the pseudo-AFM image corresponding to the updated model to the target AFM image and compare it to the previously calculated SSIM between candidate and target AFM images. The change in SSIM is again converted to a probability value to determine whether the updated model (in 3D) is accepted or rejected. The flowchart of our MC algorithm is given in Supplementary Figure 2, and the details of parameters used in Monte-Carlo algorithms are discussed in Supplementary Section 3. As shown in Figure 3, sampling runs started from both R and S models converge similarly with SSIM reaching a high value. In addition, the models accepted during the sampling become similar in 3D to our final reconstructed model.

In the above two-step MC algorithm, the scoring function for the first step is defined from the change in empirical restraints while the scoring function for the second step is defined from the change in SSIM. Therefore, in principle, the temperature parameters associated with each step should be different. To avoid this, we multiplied SSIM by 10000 and use identical temperature parameters for both steps. With this simplification, we could use one annealing scheme (Supplementary Figure 3A) modifying the temperature between 0.2 and 1.0. In Supplementary Figure 3B, we show how scores change in first and second MC steps with accepted steps for one of the trajectories, demonstrating our first MC step is more restrictive in rejecting new model than the second MC step, however, the ranges of change in score are comparable.

### Selecting Representative Models for an AFM Image

The structure optimization process is run in two phases. In the first phase, for a given AFM image we ran twenty trajectories in parallel each for 1.5 × 10^6^ steps, where ten trajectories randomly seeded were initiated from model R and ten trajectories were initiated from model S. At the end of the sampling, we select a few best models (based on a quantile value of 0.2 for SSIM over all the models) from each set of ten trajectories. The models from R and the models from S are compared pair-wise by calculating 3D correlation coefficients after rigid-body fitting of the kernel centers to identify which of the two models initiated from R or S are most similar, hence the most converged solutions starting from two different initial models.

In the second phase, we start from these converged solutions and continue for additional 1.5 × 10^6^ steps for twenty trajectories, ten from the R converged model obtained previously and ten from S converged model, all randomly seeded. At the end, we repeat the above analysis to determine final converged models, providing us with two solutions for a given AFM image. In total, one optimization process for one AFM image with two phases takes about 166 h.

### Comparison of Models in 3D

Two measures were used to compare the Gaussian mixture 3D models (Kawabata, 2008). In one approach, we performed a rigid-body fitting using the kernel centers between the two models, followed by computation of correlation coefficient. We use an in-house python script to perform rigid-body fitting (Diamond, 1988), and then the “gmconvert” program is used to compute the correlation coefficient (Kawabata, 2008). We also perform rigid-body fitting with UCSF Chimera using a full density distribution, where densities are computed according to a Gaussian mixture model (Cheng et al., 2015). The resulting correlation coefficients are referred to as CC3D_density_.

We also clustered potential candidate solutions to understand their conformational landscape (Supplementary Table 5). Gaussian mixture models are converted into voxel-based density maps and principal component analysis of the set of maps was performed. The voxel-based density maps are then projected into a lower 2-dimensional space defined by the first two principal components. Then we performed clustering in the lower dimensional space by DBSCAN algorithm using scikit-learn. For clustering, we have used “min_sample” value of 4, and the “eps” parameter is optimized by visually checking the clustering solution. For each cluster, we determined the median conformation from the PCA projection. The clusters are ranked in terms of their size (Table 1, Supplementary Table 5).

**TABLE 1:** Details of representative models for AFM images of different class.

AFM image annotation	Image index (see Figure 1)	Top rank pairwise correlation-coefficient between two sets of candidates (CC3D_density_)	Details of representative model from R			Details of representative model from S		
			SSIM	Cluster	Similarity from median	SSIM	Cluster	Similarity from median
Open	1	0.9578	8,316.7	6	0.9885	8,315.2	6	0.9883
Open	2	0.9708	8,000.2	1	0.9702	8,038.6	3	0.9941
Open	3	0.8824	8,571.0	4	0.9802	8,584.2	2	0.9869
Spiral	4	0.9113	8,467.6	5	0.9939	8,770.2	1	0.9662
Spiral	5	0.8567	8,644.5	4	0.9824	8,669.8	1	0.9758
Spiral	6	0.9118	8,641.7	1	0.9714	8,467.6	4	0.99
Closed	7	0.978	8,789.9	1	0.9603	8,797.4	2	0.978
Closed	8	0.9327	8,644.4	2	0.9773	8,597.2	6	0.9848
Closed	9	0.9011	8,578.2	3	0.9777	8,528.8	1	0.9987
Half-spiral	10	0.8441	8,495.5	1	0.9726	8,447.5	2	0.9616
Half-spiral	11	0.8294	8,752.8	1	0.9871	8,384.8	5	0.9971
Half-spiral	12	0.8742	8,545.3	2	0.9948	8,566.4	2	0.9882

## Results

### Dataset of AFM Images and Conformations of ClpB Observed in AFM Experiment

The dataset of AFM images of ClpB molecules used in this work was previously described (Uchihashi et al., 2018). The conformations observed in the HS-AFM experiments were classified into four categories—*open, spiral, closed, and half-spiral*, with spiral being the major conformation. The detail method of defining such conformations from 2D AFM analysis is explained in the method section of the reference (Uchihashi et al., 2018). Briefly, the open conformations were identified from a histogram of “circularity” (defined from the ratio of the perimeter and area outlining each molecule). From the rest of the conformations, closed, spiral, and half-spiral conformations were identified by analyzing the height profile along the top surface of the ring. In current study we have used the annotations of conformational classes used in the above reference. The full dataset of the above AFM images consisted of 340 images. We randomly select twelve AFM images, taking three AFM images for each type of conformation (Figure 1). The AFM image dimensions are 66 × 42 (width x height) pixels, where each pixel along *x*-direction (width) is 4.545 Å and that along *y*-direction (height) is 5.477 Å. By manual inspection, we define masks for the selected AFM images over a region of interest that includes the ClpB oligomer. The preprocessing of the AFM images is discussed in detail in the Supplementary Section 2. Background corrected masked AFM images (Supplementary Table 1) were used as input for the 3D modelling problem.

In the ClpB hexameric structure, a prominent feature, seam, between two neighboring monomers is observed in spiral and close conformations (Figure 1). In open conformation, two neighboring monomers along the seam are separated. The half-spiral conformation is one of the unique conformations detected in the AFM experiment, for which the hexamer can be understood as a dimer of trimer or hexamer with two farthest seams (Uchihashi et al., 2018). Because such classes are based on 2D height data from AFM images, we aim to recover the corresponding full 3D information (Figure 1). For all AFM image classes, we begin MC sampling against a given AFM image from one of the two initial models—R (ring closed symmetric conformation based on 5OG1) or S (spiral asymmetric conformation based on 5KNE). The correlation coefficient between the two initial models, CC3D_density_, is 0.72.

### Reconstruction of *Open* Class AFM Images

**FIGURE 4:**
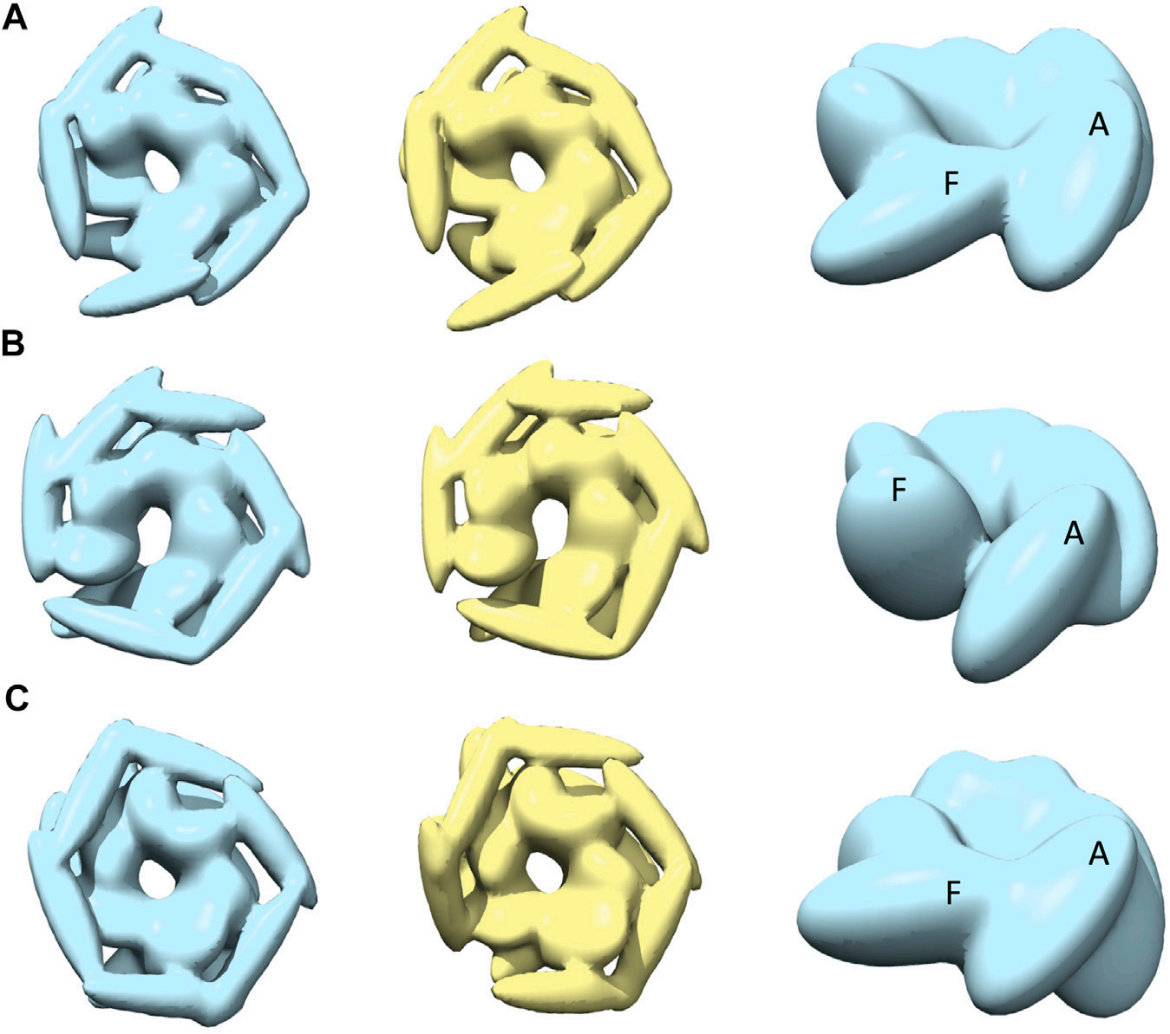
The most converged model between structural modelling starting from R and S models for open class AFM images. The rows **(A–C)** are for AFM images 1, 2 and 3, respectively. The left column shows candidate solutions from R (light blue) and middle column shows candidate solutions from S (yellow). In the right column, only the body kernel is shown from R candidate solutions given in the left column. Chains A, F are indicated on top of the kernels.

After MC sampling against open class images (AFM image 1, 2, and 3), the CC3D_density_ between models derived from R and S increase up to 0.97 for AFM image 2, 0.96 for AFM image 1, and 0.88 from AFM image 3 (see Table 1). The similarity between the AFM image and pseudo-AFM image generated from a representative model is also converged as the values from R and S solutions are similar. In addition, the converged representative models are almost identical (∼0.97 CC3D_density_ or better) to the medoids of the cluster they belong to (Table 1; Supplementary Figure 5B). Even though models are converged, due to the low-resolution nature of AFM images, some conformational differences are apparent. For example, both candidate models obtained either from R or S against AFM images 1 and 2 show a clearer separation around the original seam than in AFM image 3 (Figure 4). The body kernel arrangements for chains A and F are also different. Finally, for AFM images 1 and 3, while the body kernels orientations are similar, the coiled-coil domain orientations are different (Supplementary Figure 6). These results indicate that conformational diversity can exist even within the open state.

### Reconstruction of *Spiral* Class AFM Images

**FIGURE 5:**
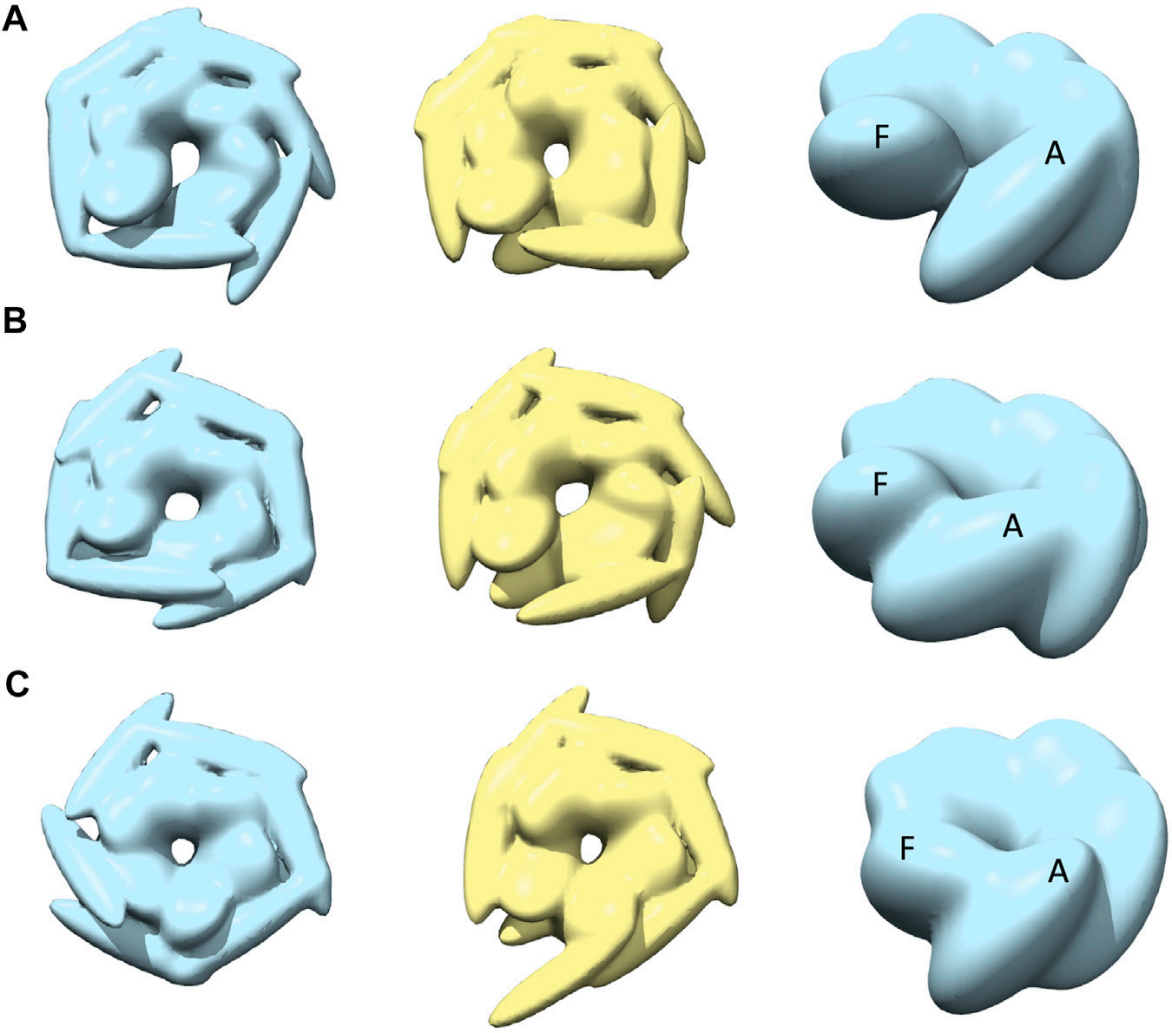
The most converged model between structural modelling starting from R (light blue) and S (yellow) for spiral class AFM images. The rows **(A–C)** are for AFM images 4, 5 and 6, respectively.

The best-converged models against the spiral class AFM images are shown in Figure 5 and details of their similarities are given in Table 1. The convergence of the models is high for AFM images 4 and 6 (CC3D_density_ >0.91). For AFM images 4 and 5, models derived from R show a hexameric arrangement (Figures 5A,B, in the left), which is less prominent in models derived from S. In addition, the S derived models show a clear separation around the seam between chains A and F (Figures 5A,B, in the middle). The arrangement of head kernels in all the models derived from R is similar to the blades of a propeller (Carroni et al., 2014), where one end is connected to the AAA1+ domain and the distal end is pointing towards a neighboring AAA1+ domain. Regarding the body kernels, their orientations around the seam show some diversity (Figure 5, right). More specifically, for the R model of AFM image 6, chains A and F are interacting through AAA1+ domain residues (residues 160 and 330) (Figure 5C, left and right), whereas for AFM images 4 and 5, chain A AAA2+ domain residues (residues 560–750) are interacting with chain F AAA1+ domain (Figures 5A,B, more evident from S models shown in the middle). Therefore, the spiral nature of the conformations is clearly exhibited in the models obtained against AFM images 4 and 5; a twist in the orientation of body kernels resulted in a vertical shift in an upward direction for chain F from chain A. Such a twist does not appear in the model obtained against AFM image 6. However, in this case, a spiral feature can be seen from head kernels, showing one end of the head kernel from the chain F is more upward than the head kernel from chain A.

### Reconstruction of *Closed* Class AFM Images

**FIGURE 6:**
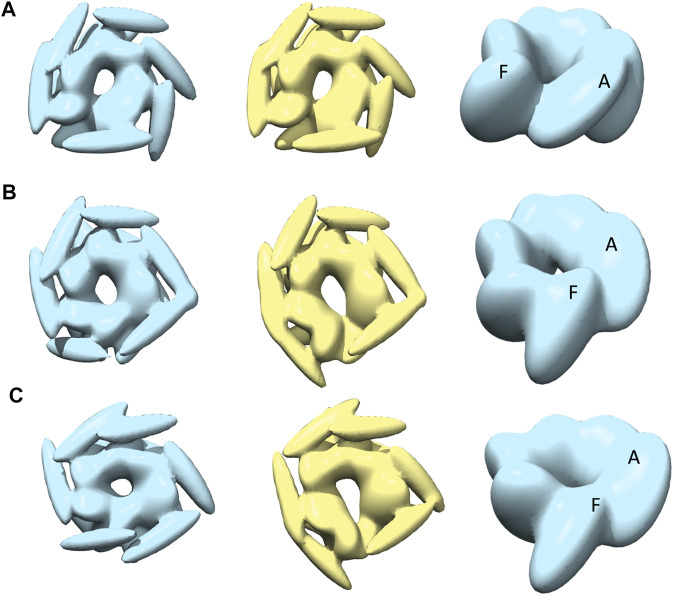
The most converged model between structural modelling starting from R (light blue) and S (yellow) for closed class AFM images. The rows **(A–C)** are for AFM images 7, 8 and 9, respectively.

The best-converged models against closed class AFM images are shown in Figure 6. In particular, for AFM image 7, we obtained the best-converged results out of all 12 AFM images (0.978) (Table 1). The convergence is also high for AFM images 8 and 9 (>0.90). Note that for such AFM images a pre-rotation of the initial model along the *z*-axis to match the seam observed in the AFM image was needed and as a result, the whole system is rotated (compared to the result for AFM image 7) (Figure 6).

The closed nature of the conformation as observed in AFM images (Figure 1) is clearer in the case of R-derived models (Figure 6, leftmost column), with chains A and F tightly closed and more parallel to each other. However, the interaction between chains A and F, in R-derived models, is different for AFM image 7, compared to AFM images 8 and 9, indicating conformational heterogeneity among the closed state. For AFM image 7, the chain A body kernel region hosting the AAA2+ domain is interacting with the chain F body kernel region hosting the AAA1+ domain (Figure 6, rightmost column), while the orientations of chain A and F body kernels for AFM images 8 and 9 are more slanted and parallel (Figures 6B,C, in the right). In addition, chains A, E and F head kernels are laying horizontally in the XY-plane and no interaction is observed between the distal end of chains B, C, and D head kernels as they are oriented downward keeping a parallel placement (Figure 6A, left). For AFM image 8, the placement of the chain F head kernel is different (distal end facing downward) from the other head kernels (Figure 6B, left), and for AFM image 9, head kernels are arranged following a hexameric symmetry, similar to the blades of a fan (Figure 6C, left).

While the representative models derived from S are less compact than those from R, their 2D similarities to the target AFM image are similar. A more open-like structure is seen for models derived from S (AFM images 8 and 9), where chains are separated at the seam (Figures 6B,C in the middle). In this case, the chain A tail kernel is pointing towards the neighboring AAA2+ domain from chain F. Such an interaction, between the C-terminal domain and AAA2+ domain indicates an alternative way of forming the closed hexamer. The diversity of resulting models indicates a possible conformational heterogeneity within closed state structures, which is difficult to solely characterize from the top view experiments by AFM.

Finally, we should also note that the arrangement of the head kernels differs from the models obtained in other classes. For spiral class AFM images, head kernels are oriented to form a hexameric arrangement (Figure 5), which is quite different from the model for the closed AFM images where coiled-coiled domains are oriented further away from each other (Figure 6).

### Reconstruction of *Half-Spiral* Class AFM Images

**FIGURE 7:**
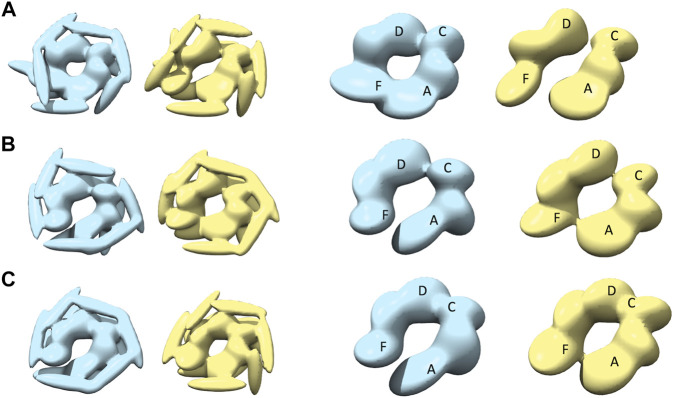
The most converged model between structural modeling starting from R (light blue) and S (yellow) for half-spiral class AFM images. The rows **(A–C)** are for AFM images 10, 11, and 12, respectively. In the third and fourth columns, only the body kernel are shown from R and S candidate solutions given in the first and second columns, respectively. The chains A, F, C, and D are shown on top of the kernels.

Modeling against the half-spiral class AFM images was also performed (Figure 7 and Table 1). In all the cases, two opened seams are observed with additional seams between chains C and D (see Figures 7A–C). However, the maximum similarities between best models are lower than for other target AFM images (>0.8), indicating that the modeling of the half-spiral state is more complex. More specifically, S derived models show more open-like conformations than R derived models. The best convergence among half-spiral cases is obtained for AFM image 12 (Figure 7C), in which the seam between C and D is less prominent than between chain A and F. These models provide insights into the possible overall arrangements of the hexamer chains in the half-spiral conformational state.

### Conformational Variations within Models Other than Converged Models

To assess conformational variations of the models for a particular target AFM image, we also examined other candidate models using clustering (Materials and Methods *Comparison of Models in 3D* and Supplementary Table 5) in addition to the most converged model. For example, we compared a converged representative model to the median of the cluster to which it belongs. In most cases, the converged model is very similar to the cluster median (>0.95 CC3D_density_, Supplementary Figure 5B). We also checked the similarity of the converged representative models to the median of the largest cluster which may not include the representative model (Supplementary Figure 5A) and found high similarity in most of the cases. We observed that the conformational variation is mostly due to the orientation of the flexible coiled-coil domain and the positioning of the tail-kernels in relation to the body kernel around the seam (Supplementary Table 6). This confirms that our converged models capture the hexameric arrangement robustly with finer differences arising due to the flexible coiled-coil region. In addition, we analyzed the similarity between initial conformations and final reconstructed models to characterize the class of such conformations (Supplementary Figure 7). The MC sampling generates models significantly different from the initial model which indicates that our sampling generates novel models based on the respective AFM images. This is particularly true for some half-spiral models that show the presence of two seams (Figure 7).

### Interpolated Conformational Transition between Different Converged Models

The HS-AFM imaging clearly demonstrated that ClpB undergoes large conformational changes. In the current study, we did not aim to recover the mechanism of such conformational change. However, it is possible to compare two reconstructed models from different AFM image classes and visualize such transitions by interpolation between volume models using Chimera “morph map” feature (Pettersen et al., 2004; Meng et al., 2006). We have used the body kernel representation (from the R model) of the four most converged reconstructions, namely AFM images 1 (open), 2 (open), 6 (spiral), and 7 (closed). For the spiral class, we observe chains A and F along the seam are oriented in a slanted way such that the AAA1+ domain in the body kernel from chain F is interacting with the AAA1+ domain from chain A. However, for the closed class AFM image, the orientation of the body kernel from chain A slips relative to chain F, and its AAA2+ domain interacts with the AAA1+ domain of chain F. Such a movement is clearly visualized in the Supplementary Movie 1. Similarly, we compare the orientations of body kernels for the closed class AFM image against the open class model. In the open class AFM image 2, chains A and F are separated while keeping a similar orientation as observed in AFM image 7 (closed). This also can be seen in the interpolated visualization (Supplementary Movie 2). AFM image 1 is also an open class image, however, in this case, the body kernel from chain F is more slanted than in AFM image 2; the AAA1+ domain from chain F is pointing towards the AAA2+ domain of chain A. A comparison of spiral class AFM image and open class AFM image 1 indicates that the body kernel from chain F is getting separated while keeping a slanted orientation (Supplementary Movie 3). Lastly, morphing of the model generated from AFM image 2 (open) into the model generated from AFM image 6 (spiral) shows that the orientations of chain A and F remain rather similar (Supplementary Movie 4).

## Discussion

HS-AFM images of biomolecules can include a wealth of information on various conformational states, yet 3D reconstructed models can provide further functional implications. To this aim, we propose a novel method for reconstructing 3D models using MC sampling based on a coarse-grained representation—the Gaussian mixture model. We applied our method to reconstruct the hexameric structure of *T. thermophilus* ClpB protein as observed in the HS-AFM experiment conducted in presence of ATP (Uchihashi et al., 2018).

The basic algorithm applied to a few theoretical systems was previously published (Dasgupta et al., 2020). Here we further extend our algorithm, in particular, we adopted a state-of-the-art 2D image comparison technique—SSIM for quantifying the agreement between the 3D model and AFM images. Our previous restraint scheme was also improved by introducing a new harmonic restraint scheme built from the interpolation of the correlation coefficient from two different initial models. This extension was necessary because the HS-AFM experiments observed ClpB conformational dynamics for a much wider timescale, and as a result, some conformations could not be characterized by any of the known conformational states (Uchihashi et al., 2018). For example, while spiral arrangements are frequently observed in the HS-AFM experiment, the overall conformation is different from the known asymmetric spiral conformation (Deville et al., 2017). Therefore, 3D modeling against AFM images was performed from the two known conformational states that capture some of ClpB conformational diversity in terms of symmetric/asymmetric systems. In addition, in the harmonic restraining scheme, we treat attraction and repulsion in a separate way (Supplementary Section 4), with stronger repulsive interactions to eliminate any severe steric clash between the 3D ellipsoidal kernels. We also used a connectivity restraint between kernels from the same monomer (Supplementary Figure 4) to ensure that these kernels, within a monomer, remain connected behaving as hinges between a pair of kernels during random moves.

Models with good convergence were obtained for three AFM image classes—open, closed and spiral. The open and spiral classes show highly conserved 3D models (Table 1), and for the closed class we obtained fairly conserved reconstructions. However, for the half-spiral class, modeling was more challenging due to a characteristic feature in this class—an additional seam between chains C and D. One structural feature of ClpB important during model constructions is the presence of a seam between chains A and F. The initial conformation S has a prominent seam and important for the modeling for the spiral models from the AFM images. However, for closed class of AFM images, the models derived from R (without a prominent seam) show more consistent results. In the case of half-spiral states, there are two seams, and therefore such conformations are more distinct from either of the initial conformations. This is likely to be a reason for the difficulty to model half-spiral forms. Nonetheless, the initial correlation between two models (based on 5OG1 and 5KNE) was 0.72 which significantly increased during the modeling to more than 0.82 even for half-spiral AFM images. Moreover, the similarity between initial models and the final conformations are low for most of the cases (Supplementary Figure 7). These results demonstrate that our algorithm with coarse-grained model can sample models far from the initial models.

To examine the overall distributions and relations between reconstructed models predicted against different AFM images, the cross-correlation between the 3D models were calculated (Tables 2, 3). These data show that the models reconstructed against the same class of AFM images are more similar to each other than for other classes, i.e., the models are capturing the essential details of AFM experimental observation. On the other hand, it can be observed that cross-correlations between models for closed and spiral AFM image classes are moderately high (>0.81), indicating that the conformations of these two classes are similar. The open conformation can have considerable different hexameric arrangements, which is illustrated from lower cross-correlations within the open class AFM images.

**TABLE 2:** Cross-correlation (CC3D_density_) between converged representative models for each AFM image derived from R and S models. The entries above the diagonal show similarity between reconstructed models derived from the R model, and those below the diagonal show similarity between reconstructed models derived from the S model.

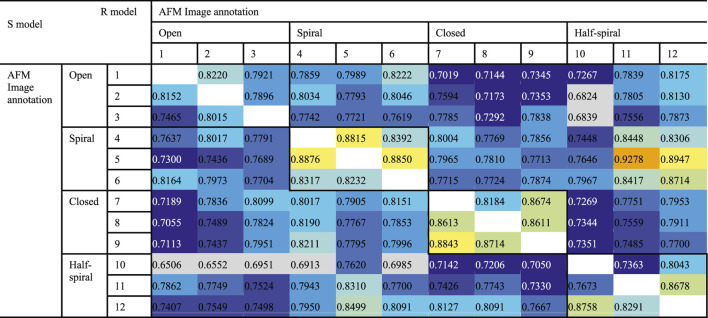

**TABLE 3:** Summary of Table 2 to average cross-correlation between different types of AFM images (by averaging over each 3 × 3 block in Table 2).

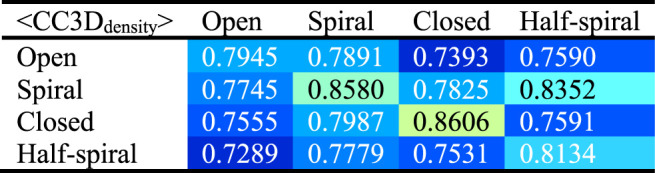

While we focused on the analysis of conformational dynamics of ClpB in this study, we developed our algorithms aiming to apply to other AFM studies. In the current implantation we need at least some knowledge of the protein under investigation and how many domains we should expect. Also, we need to know rough size and shape of those domains. Since our approach is based on coarse-grained model, information from homologous structures can be utilized as we used the homologous structures (5OG1 and 5KNE) in this study. The algorithm can be theoretically applied in a completely *ab-initio* manner; however, such a detailed evaluation is yet to be done.

To conclude, in the present work, we applied a hybrid modeling approach to provide further interpretation of experimental HS-AFM images of bacterial ClpB. The structural dynamics of ClpB is crucial to understand how a healthy cell maintains its proper functioning. Previously, the HS-AFM experiment was performed without substrate in presence of ATP, and analysis of 2D images revealed novel conformational classes of the ClpB oligomer. Our current study using hybrid modeling from AFM data enabled us to reconstruct 3D models for each of the conformational classes. Such models show the full hexameric architecture of ClpB including all domains related to disaggregation and capture specific features of the four conformational states observed in the AFM images. In particular, some novel conformational classes were suggested, such that the open class could be divided into sub-classes. In addition, conformational transitions between 3D models representing the different classes were obtained. More specifically, we observed that a slipping motion between two monomers around the seam in spiral conformation might be necessary to reach the closed conformation. We have used a coarse-grained representation for ClpB structure in line with the low-resolution nature of the AFM data and thus a detailed atomic-level picture is not directly obtained from our analysis. However, our study provides domain-level 3D structural information with structural insights into the different types of ClpB hexameric arrangements observed in the HS-AFM experiments. While the present work aimed to address growing attention on the function of ClpB disaggretase, it also demonstrated the usability of our algorithm, hybrid modeling based 2D AFM images, on HS-AFM experimental data. In the future, such applications could help to address questions regarding the structure, dynamics, and function of biomolecules from HS-AFM experiments.

## Data Availability

The raw data supporting the conclusions of this article will be made available by the authors upon request.

## References

[B1] Amyot R. Flechsig H. (2020). BioAFMviewer: An Interactive Interface for Simulated AFM Scanning of Biomolecular Structures and Dynamics. Plos Comput. Biol. 16, e1008444. 10.1371/journal.pcbi.1008444 33206646 PMC7710046

[B2] Barnett M. E. Nagy M. Kedzierska S. Zolkiewski M. (2005). The Amino-Terminal Domain of ClpB Supports Binding to Strongly Aggregated Proteins. J. Biol. Chem. 280, 34940–34945. 10.1074/jbc.M505653200 16076845

[B3] Carroni M. Kummer E. Oguchi Y. Wendler P. Clare D. K. Sinning I. (2014). Head-to-tail Interactions of the Coiled-Coil Domains Regulate ClpB Activity and Cooperation with Hsp70 in Protein Disaggregation. Elife 3, e02481. 10.7554/eLife.02481 24843029 PMC4023160

[B4] Chen P.-c. Shevchuk R. Strnad F. M. Lorenz C. Karge L. Gilles R. (2019). Combined Small-Angle X-ray and Neutron Scattering Restraints in Molecular Dynamics Simulations. J. Chem. Theor. Comput. 15, 4687–4698. 10.1021/acs.jctc.9b00292 31251056

[B5] Cheng A. Henderson R. Mastronarde D. Ludtke S. J. Schoenmakers R. H. M. Short J. (2015). MRC2014: Extensions to the MRC Format Header for Electron Cryo-Microscopy and Tomography. J. Struct. Biol. 192, 146–150. 10.1016/j.jsb.2015.04.002 25882513 PMC4642651

[B6] Dasgupta B. Miyashita O. Tama F. (2020). Reconstruction of Low-Resolution Molecular Structures from Simulated Atomic Force Microscopy Images. Biochim. Biophys. Acta Gen. Subj. 1864, 129420. 10.1016/j.bbagen.2019.129420 31472175

[B7] Degiacomi M. T. Schmidt C. Baldwin A. J. Benesch J. L. P. (2017). Accommodating Protein Dynamics in the Modeling of Chemical Crosslinks. Structure 25, 1751–1757. 10.1016/j.str.2017.08.015 28966018

[B8] Derevyanko G. Grudinin S. (2014). HermiteFit: Fast-Fitting Atomic Structures into a Low-Resolution Density Map Using Three-Dimensional Orthogonal Hermite Functions. Acta Cryst. D Biol. Crystallogr. 70, 2069–2084. 10.1107/S1399004714011493 25084327

[B9] Deville C. Carroni M. Franke K. B. Topf M. Bukau B. Mogk A. (2017). Structural Pathway of Regulated Substrate Transfer and Threading through an Hsp100 Disaggregase. Sci. Adv. 3, e1701726. 10.1126/sciadv.1701726 28798962 PMC5544394

[B10] Deville C. Franke K. Mogk A. Bukau B. Saibil H. R. (2019). Two-Step Activation Mechanism of the ClpB Disaggregase for Sequential Substrate Threading by the Main ATPase Motor. Cell Rep. 27, 3433–3446. 10.1016/j.celrep.2019.05.075 31216466 PMC6593972

[B11] Diamond R. (1988). A Note on the Rotational Superposition Problem. Acta Cryst. Sect. A. 44, 211–216. 10.1107/s0108767387010535

[B12] Doyle S. M. Genest O. Wickner S. (2013). Protein rescue from Aggregates by Powerful Molecular Chaperone Machines. Nat. Rev. Mol. Cel. Biol. 14, 617–629. 10.1038/nrm3660 24061228

[B13] Ekimoto T. Ikeguchi M. (2018). Hybrid Methods for Modeling Protein Structures Using Molecular Dynamics Simulations and Small-Angle X-Ray Scattering Data. Adv. Exp. Med. Biol. 1105, 237–258. 10.1007/978-981-13-2200-6_15 30617833

[B14] Faini M. Stengel F. Aebersold R. (2016). The Evolving Contribution of Mass Spectrometry to Integrative Structural Biology. J. Am. Soc. Mass. Spectrom. 27, 966–974. 10.1007/s13361-016-1382-4 27056566 PMC4867889

[B15] Fuchigami S. Niina T. Takada S. (2021). Case Report: Bayesian Statistical Inference of Experimental Parameters via Biomolecular Simulations: Atomic Force Microscopy. Front. Mol. Biosci. 8, 636940. 10.3389/fmolb.2021.636940 33778008 PMC7987833

[B16] Gates S. N. Yokom A. L. Lin J. Jackrel M. E. Rizo A. N. Kendsersky N. M. (2017). Ratchet-like Polypeptide Translocation Mechanism of the AAA+ Disaggregase Hsp104. Science 357, 273–279. 10.1126/science.aan1052 28619716 PMC5770238

[B17] Glover J. R. Lindquist S. (1998). Hsp104, Hsp70, and Hsp40: A Novel Chaperone System that Rescues Previously Aggregated Proteins. Cell 94, 73–82. 10.1016/s0092-8674(00)81223-4 9674429

[B18] Gorba C. Tama F. (2010). Normal Mode Flexible Fitting of High-Resolution Structures of Biological Molecules Toward SAXS Data. Bioinform. Biol. Insights 4, 43–54. 10.4137/bbi.s4551 20634984 PMC2901630

[B19] Grubisic I. Shokhirev M. N. Orzechowski M. Miyashita O. Tama F. (2010). Biased Coarse-Grained Molecular Dynamics Simulation Approach for Flexible Fitting of X-ray Structure into Cryo Electron Microscopy Maps. J. Struct. Biol. 169, 95–105. 10.1016/j.jsb.2009.09.010 19800974

[B20] Haslberger T. Bukau B. Mogk A. (2010). Towards a Unifying Mechanism for ClpB/Hsp104-Mediated Protein Disaggregation and Prion propagation. Biochem. Cell Biol. 88, 63–75. 10.1139/o09-118 20130680

[B21] Kawabata T. (2018). Gaussian-input Gaussian Mixture Model for Representing Density Maps and Atomic Models. J. Struct. Biol. 203, 1–16. 10.1016/j.jsb.2018.03.002 29522817

[B22] Kawabata T. (2008). Multiple Subunit Fitting into a Low-Resolution Density Map of a Macromolecular Complex Using a Gaussian Mixture Model. Biophys. J. 95, 4643–4658. 10.1529/biophysj.108.137125 18708469 PMC2576401

[B23] Kim D. N. Moriarty N. W. Kirmizialtin S. Afonine P. V. Poon B. Sobolev O. V. (2019). Cryo_fit: Democratization of Flexible Fitting for Cryo-EM. J. Struct. Biol. 208, 1–6. 10.1016/j.jsb.2019.05.012 31279069 PMC7112765

[B24] Lee S. Choi J.-M. Tsai F. T. F. (2007). Visualizing the ATPase Cycle in a Protein Disaggregating Machine: Structural Basis for Substrate Binding by ClpB. Mol. Cell 25, 261–271. 10.1016/j.molcel.2007.01.002 17244533 PMC1855157

[B25] Malhotra S. Träger S. Dal Peraro M. Topf M. (2019). Modelling Structures in Cryo-EM Maps. Curr. Opin. Struct. Biol. 58, 105–114. 10.1016/j.sbi.2019.05.024 31394387

[B26] Martí-Renom M. A. Stuart A. C. Fiser A. Sánchez R. Melo F. Sali A. (2000). Comparative Protein Structure Modeling of Genes and Genomes. Annu. Rev. Biophys. Biomol. Struct. 29, 291–325. 10.1146/annurev.biophys.29.1.291 10940251

[B27] Meng E. C. Pettersen E. F. Couch G. S. Huang C. C. Ferrin T. E. (2006). Tools for Integrated Sequence-Structure Analysis with UCSF Chimera. BMC Bioinform. 7, 339. 10.1186/1471-2105-7-339 PMC1570152 16836757

[B28] Miyashita O. Kobayashi C. Mori T. Sugita Y. Tama F. (2017). Flexible Fitting to Cryo-EM Density Map Using Ensemble Molecular Dynamics Simulations. J. Comput. Chem. 38, 1447–1461. 10.1002/jcc.24785 28370077

[B29] Mizuno S. Nakazaki Y. Yoshida M. Watanabe Y.-h. (2012). Orientation of the Amino-Terminal Domain of ClpB Affects the Disaggregation of the Protein. FEBS J. 279, 1474–1484. 10.1111/j.1742-4658.2012.08540.x 22348341

[B30] Mogk A. Bukau B. Kampinga H. H. (2018). Cellular Handling of Protein Aggregates by Disaggregation Machines. Mol. Cell 69, 214–226. 10.1016/j.molcel.2018.01.004 29351843

[B31] Mogk A. Kummer E. Bukau B. (2015). Cooperation of Hsp70 and Hsp100 Chaperone Machines in Protein Disaggregation. Front. Mol. Biosci. 2, 22. 10.3389/fmolb.2015.00022 26042222 PMC4436881

[B32] Mogk A. Schlieker C. Strub C. Rist W. Weibezahn J. Bukau B. (2003). Roles of Individual Domains and Conserved Motifs of the AAA+ Chaperone ClpB in Oligomerization, ATP Hydrolysis, and Chaperone Activity. J. Biol. Chem. 278, 17615–17624. 10.1074/jbc.M209686200 12624113

[B33] Nagai T. Mochizuki Y. Joti Y. Tama F. Miyashita O. (2018). Gaussian Mixture Model for Coarse-Grained Modeling from XFEL. Opt. Express 26, 26734. 10.1364/OE.26.026734 30469754

[B34] Nakano M. Miyashita O. Jonic S. Tokuhisa A. Tama F. (2018). Single-particle XFEL 3D Reconstruction of Ribosome-Size Particles Based on Fourier Slice Matching: Requirements to Reach Subnanometer Resolution. J. Synchrotron Radiat. 25, 1010–1021. 10.1107/S1600577518005568 29979162

[B35] Niina T. Fuchigami S. Takada S. (2020). Flexible Fitting of Biomolecular Structures to Atomic Force Microscopy Images via Biased Molecular Simulations. J. Chem. Theor. Comput. 16, 1349–1358. 10.1021/acs.jctc.9b00991 31909999

[B36] Pettersen E. F. Goddard T. D. Huang C. C. Couch G. S. Greenblatt D. M. Meng E. C. (2004). UCSF Chimera? A Visualization System for Exploratory Research and Analysis. J. Comput. Chem. 25, 1605–1612. 10.1002/jcc.20084 15264254

[B37] Rizo A. N. Lin J. Gates S. N. Tse E. Bart S. M. Castellano L. M. (2019). Structural Basis for Substrate Gripping and Translocation by the ClpB AAA+ Disaggregase. Nat. Commun. 10, 2393. 10.1038/s41467-019-10150-y 31160557 PMC6546751

[B38] Rout M. P. Sali A. (2019). Principles for Integrative Structural Biology Studies. Cell 177, 1384–1403. 10.1016/j.cell.2019.05.016 31150619 PMC6810593

[B39] Schindler C. E. M. de Vries S. J. Sasse A. Zacharias M. (2016). SAXS Data Alone Can Generate High-Quality Models of Protein-Protein Complexes. Structure 24, 1387–1397. 10.1016/j.str.2016.06.007 27427479

[B40] Srivastava A. Tiwari S. P. Miyashita O. Tama F. (2020). Integrative/Hybrid Modeling Approaches for Studying Biomolecules. J. Mol. Biol. 432, 2846–2860. 10.1016/j.jmb.2020.01.039 32061933

[B41] Tokuhisa A. Jonic S. Tama F. Miyashita O. (2016). Hybrid Approach for Structural Modeling of Biological Systems from X-ray Free Electron Laser Diffraction Patterns. J. Struct. Biol. 194, 325–336. 10.1016/j.jsb.2016.03.009 26972893

[B42] Trabuco L. G. Villa E. Mitra K. Frank J. Schulten K. (2008). Flexible Fitting of Atomic Structures into Electron Microscopy Maps Using Molecular Dynamics. Structure 16, 673–683. 10.1016/j.str.2008.03.005 18462672 PMC2430731

[B43] Uchihashi T. Watanabe Y.-H. Nakazaki Y. Yamasaki T. Watanabe H. Maruno T. (2018). Dynamic Structural States of ClpB Involved in its Disaggregation Function. Nat. Commun. 9, 2147. 10.1038/s41467-018-04587-w 29858573 PMC5984625

[B44] Wang Z. Bovik A. C. (2009). Mean Squared Error: Love it or Leave it? A New Look at Signal Fidelity Measures. IEEE Signal. Process. Mag. 26, 98–117. 10.1109/MSP.2008.930649

[B45] Wang Z. Bovik A. C. Sheikh H. R. Simoncelli E. P. (2004). Image Quality Assessment: from Error Visibility to Structural Similarity. IEEE Trans. Image Process. 13, 600–612. 10.1109/tip.2003.819861 15376593

[B46] Webb B. Sali A. (2014). Comparative Protein Structure Modeling Using MODELLER. Curr. Protoc. Bioinform. 47, 5.6.1–5.6.32. 10.1002/0471250953.bi0506s47 25199792

[B47] Weibezahn J. Tessarz P. Schlieker C. Zahn R. Maglica Z. Lee S. (2004). Thermotolerance Requires Refolding of Aggregated Proteins by Substrate Translocation through the central Pore of ClpB. Cell 119, 653–665. 10.1016/j.cell.2004.11.027 15550247

[B48] Yokom A. L. Gates S. N. Jackrel M. E. Mack K. L. Su M. Shorter J. (2016). Spiral Architecture of the Hsp104 Disaggregase Reveals the Basis for Polypeptide Translocation. Nat. Struct. Mol. Biol. 23, 830–837. 10.1038/nsmb.3277 27478928 PMC5509435

