# Supplementary material for: Reconstruction of Three-Dimensional Conformations of Bacterial ClpB from High-Speed Atomic-Force-Microscopy Images
Source: Front Mol Biosci. 2021 Aug 5;8:704274. doi: 10.3389/fmolb.2021.704274 (PMC8376356; doi:10.3389/fmolb.2021.704274)
Supplement: Supplementary file 2 [file DataSheet1.pdf]

Supplementary Data for:

Reconstruction of Three-Dimensional Conformations of Bacterial ClpB from High-Speed Atomic-Force-Microscopy Images

Bhaskar Dasgupta[1], Osamu Miyashita[1], Takayuki Uchihashi[2,3,4], Florence Tama[1,4,5*]

Affiliations:
[1]Computational Structural Biology Research Team, RIKEN-Center for Computational Science, Hyogo, Japan
[2]Institute for Glyco-core Research (iGCORE), Nagoya University, Furo-cho, Chikusa-ku, Nagoya, Japan
[3]Exploratory Research Center on Life and Living Systems (ExCELLS), National Institutes of Natural Sciences, Okazaki, Japan
[4]Department of Physics, Graduate School of Science, Nagoya University, Nagoya, Japan
[5]Institute of Transformative Bio-Molecules, Nagoya University, Nagoya, Japan

1. Preprocessing of AFM images:

Each of the AFM images (Uchihashi et al., 2018) was first processed to define a region of interest by using a manually defined binary mask. The manual definition of binary mask is done by carefully observing an AFM image and selecting a region of ClpB structure near the center excluding adjacent molecules in an image editing software (Schneider et al., 2012). This region is termed as region of interest (ROI). The ROI includes the height information of a molecule which is subjected to modelling in our study. We calculate minimum height data inside ROI and replace height data outside ROI by the minimum value. This background corrected AFM image data is saved as 32-bit float in tiff format. The mask for 9 AFM images and corresponding background corrected AFM images are shown in Supplementary Table 1.

Supplementary Table 1: Masked AFM images after preprocessing.

AFM Image Annotation	Image Index	Masked AFM image for sampling	AFM Image Annotation	Image Index	Masked AFM image for sampling
	1	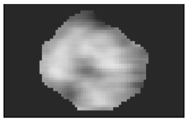		4	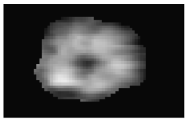
Open	2	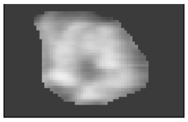	Spiral	5	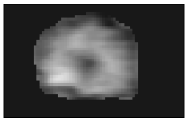
	3	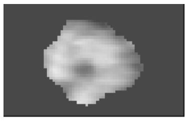		6	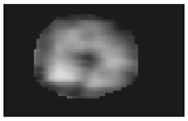
	7	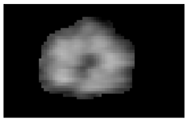		10	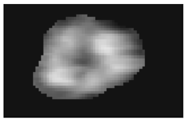
Close	8	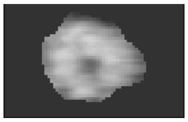	Half-spiral	11	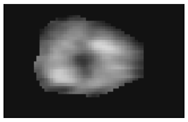
	9	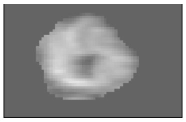		12	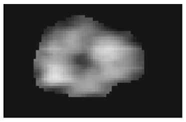

2. Preprocessing initial models before sampling:

The preparation of initial models is discussed in main text. Here, we add further information about A) superposition of monomer of ClpB to the chains in hexameric ClpB, B) coarse graining monomer chain by Gaussian mixture model, C) pre-orientation of hexameric models according to AFM images and D) setting of a clipping-plane for image comparison.

Supplementary Table 2: Superimposition statistics for fitting monomer of ClpB from 1QVR, chain A and individual chains of ClpB or HSP104 from hexamers.

Chain IDs of hexamer	Structural similarity between hexamer chains and ClpB monomer			
	5KNE		5OG1	
	Number of pruned atom pairs	RMSD over pruned pairs (Å)	Number of pruned atom pairs	RMSD over pruned pairs (Å)
A	176	1.32	107	1.31
B	229	1.28	44	1.25
C	267	1.31	114	1.17
D	283	1.33	119	1.13
E	312	1.29	84	1.15
F	222	1.35	165	1.30

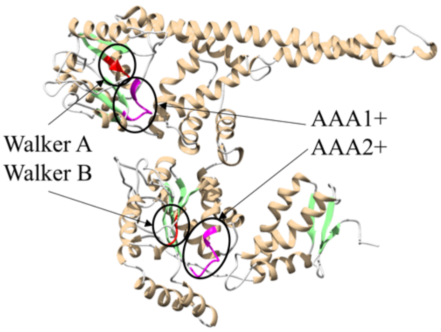

Supplementary Figure 1: The N-terminal deleted monomer of ClpB from *T. thermophilus* (1QVR, chain A). The atomic structure is colored by secondary structure (helix in tan, strands in light green and coils in gray), and four important motifs are colored in red (Walker A and B) and magenta (AAA1+ and AAA2+). The corresponding 3-kernel representation are shown in Fig 2 of main text.

A. Superposition of monomer of ClpB to the chains in hexameric ClpB:

The monomer of ClpB from *T. thermophilus* (1QVR, chain A) (Lee et al., 2003) is sequentially the same construct as in the AFM experiment, only the difference is that in the AFM experiment the N-terminal region is deleted (Uchihashi et al., 2018). We prepared hexameric models of ClpB using *T. thermophilus* primary structure, based on Yeast Hsp104 (5KNE, sequence identity to 1QVR, chain A is 44.8% (Madeira et al., 2019)) (Yokom et al., 2016) or *E. coli* ClpB (5OG1, sequence identity to 1QVR, chain A is 56.1%) (Deville et al., 2017). Chain A of 1QVR is superimposed on the individual chains of 5KNE and 5OG1 (chains A, B, C, D, E, and F) as rigid-body superposition by Chimera 'matchmaker' (Pettersen et al., 2004; Meng et al., 2006). In some of the chains in hexamer (5KNE and 5OG1) the long coiled-coil middle domain is only partially resolved or not resolved, and in other chains, the coiled-coil domains are oriented very differently than that in 1QVR, chain A. Moreover, in some cases, the N-terminal region (which is deleted in further analysis) is oriented differently. Therefore, in Chimera, we have used atom pairs pruning to locate more conserved regions. The number of pruned atom pairs and root mean square deviation between 1QVR chain A and chains in hexamer are shown in Supplementary Table 2. Moreover, because of the coarse-graining of our initial model, finer changes in atomic coordinate between ClpB monomer and hexamers are insignificant given the nanometer resolution of AFM image data.

B. Coarse graining monomer chain by Gaussian mixture model:

In order to obtain a coarse-grain representation of the hexameric ClpB, we fitted a 3-kernel Gaussian mixture model to the atomic coordinates of each monomer (after deleting N-terminal domain) superimposed to the chains A, B, C, D, E and F. The 3-kernel Gaussian mixture model is obtained from the software gmconvert with argument '-ng 3'(Kawabata, 2008) which fits 3 Gaussian kernels to the 3D coordinates by expectation-maximization algorithm. The resulting model can be represented by,

$$f(\boldsymbol{r}|\boldsymbol{\mu},\Sigma) = \sum_{i}^{N=3} w_i \varphi_i(\boldsymbol{r}|\boldsymbol{\mu_i},\Sigma_i) \quad (1)$$

Where, $\boldsymbol{r}$ represents position vector of an atom, $\boldsymbol{\mu}$ and Σ are center of mixture model and overall covariance matrix. The w_i is the weight of the i-th kernel, and $\boldsymbol{\mu_i}, \Sigma_i$ are center and covariance matrix of the i-th kernel. After all the chains are converted to 3-kernel representation, they are accumulated in a single model and weights are normalized over all the kernels giving rise to the 18-kernel representation of a hexamer. The monomer chain resulted from 1QVR chain A (where loops are already modelled, see main text) is shown in Supplementary Figure 1 (Webb and Sali, 2014), and its 3-kernel representation is shown in main text Figure 2I. In the main text Figure 2I, we represent those 3-kernels by head, body and tail as annotated. The head kernel includes mostly the long coiled-coil middle domain, and tail kernel includes mostly C-terminal domain. The body kernel includes top AAA+ domain (AAA1+) together with the linker region connecting to the bottom AAA+ domains (AAA2+). The AAA2+ domain is at the junction of body and tail kernels. The two Walker motifs are also included in the body kernel. The orientation of the part included in the body kernel guides the hexameric architecture.

C. Pre-orientation of hexameric models according to AFM images:

In Figure 2 of the main text, a seam can be observed from the top view of hexameric models, and in Figure 1 we see such a seam can also be observed in the AFM images (Uchihashi et al., 2018). However, we observed that our sampling algorithm cannot generate such a seam by randomly moving the kernels in all possible ways. This is due to the sampling algorithm has no special moves to match the seam globally. Therefore, before sampling, we rotate our initial models around the z-axis so that seam in the hexameric model (observed from top view) matches the seam in the respective target AFM image. The seams can be clearly seen for AFM image 7 (closed class) and the top view of the S model, so initially we rotate S model (also an asymmetric structure in 5KNE) to fit its seam to the AFM image by manual observation. Then we fitted R model to S by applying rigid-body transformation based on kernel centers. We save these rotated initial models as models ready for sampling (Supplementary Table 3). Therefore, for most of the AFM images (class open, closed and spiral) z-rotation of Rand S are identical. This scheme however did not work well for half-spiral cases, and for them z-rotation for R and S models are different (instead of fitting R model to S, R also rotated on the basis of visual inspection).

In addition, the initial model is shifted along z-axis to match the average heights in the masked AFM image. The average height along z-axis for initial models after translation are given in Supplementary Table 3. Note that, this is done before sampling for each AFM image.

D. Setting of clipping-plane for image comparison:

In the last pre-processing step of initial model prior to sampling, we set a clipping plane for pseudo-AFM image obtained from the models serving as a background height. This step is required because we see that range of heights in masked AFM image (29.5Å to 39.5Å (or 38.7Å excluding half-spiral)) is much narrower than height range of 3D models (61.8Å). After translating initial model

along the z-axis by average height (see above), some portion of 3D models were below 0Å. The clipping plane cannot be deduced based on masked AFM image, as the range of height in the unmasked AFM image reflects the condition of AFM experiments and the depth at which the AFM probe can sample. Thus, we first calculate change in maximum height in the target AFM image (δh) due to masking as the maximum height in an unmasked AFM image minus maximum height in the masked AFM image. Then we define clipping plane (h_{plane}) from minimum height in the masked AFM image (h_{min}) and δh by $h_{plane} = h_{min} - \delta h$, and we assume AFM probe could gauge this height in similar experimental condition. The clipping plane height is also given in Supplementary Table 3.

Supplementary Table 3: Z-rotation applied to initial models for each target AFM image

AFM Image Index	Z-Rotation of S model	Z-Rotation of R model	Average height after Z-translation	Clipping plane height
1	-4.0	-4.0	34.291	3.337
2	-4.0	-4.0	36.625	9.427
3	-4.0	-4.0	35.688	9.979
4	-6.0	-6.0	20.519	-1.946
5	0.0	0.0	23.693	-1.416
6	2.0	2.0	21.935	-1.253
7	0.0	0.0	19.987	-11.558
8	44.0	44.0	33.199	1.188
9	52.0	52.0	15.612	-6.306
10	20.0	-6.0	26.160	-1.335
11	20.0	-6.0	23.057	-4.107
12	10.0	-16.0	23.547	-1.401

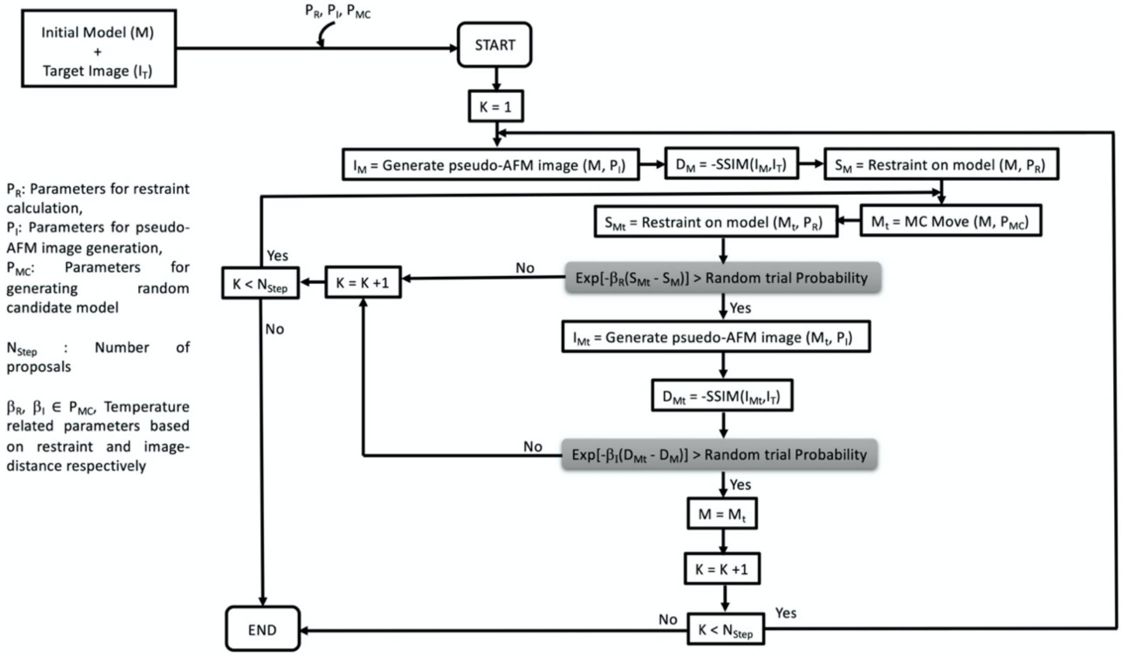

Supplementary Figure 2: Pseudocode for MC algorithm

3. **Sampling algorithm:**

The flowchart for MC algorithm is given in Supplementary Figure 2. We use a two-step MC algorithm, first on the basis of restraints of candidate model, and second on the basis of 2D image comparison technique. There are three types of parameters used to control MC process – A) parameters for restraint calculation, B) parameters related to MC move C) parameters for pseudo-AFM image generation. The choice of parameters for restraint calculation is discussed in detail in the next section.

In our MC algorithm, we use a few parameters to control width of the random move. In all the sampling, we randomly update one out of 18 kernels. To update a given kernel, we apply random translation to the kernel center and random rotation along one of the randomly chosen axes of the kernel. The width of translation is 0.5Å maximum and the width of the rotation is 0.5 degrees maximum. We proceed 1.5×10^6 steps of proposal in one run of MC process. Among these proposal steps, we apply random rigid-body translation to the 18-kernel representation in first 1000 steps. In the following proposals, kernels move independently of each other. In every 10 steps of acceptance, we saved a candidate model for subsequent analysis. The computational time required for one MC step is around 200 milliseconds and therefore for 1.5×10^6 steps for a run takes around 83 hours. However, this depends on I/O during the process, including output of model in hdf5-file, image (png-formatted) files for reference purpose, and standard output. By minimizing I/O, the time per step can be decreased to 120 milliseconds.

To generate pseudo-AFM image from coarse-grain representation of an 18-kernel model, we regard that the kernels are cut at a probability threshold giving rise to a kernel isosurface, and a sharp AFM probe interacts with these isosurface. The integration of the isosurface represents the total enclosed volume of the molecule in this representation. The shape of the kernel is included in the eigenvalues (λ_1,λ_2, and λ_3) of the covariance matrix (Σ) of the kernel and orientation of the kernel around its center ($\mathbf{r_c}$) in represented by the eigenvectors ($\mathbf{e_1}$, $\mathbf{e_2}$ and $\mathbf{e_3}$) of the Σ. The radius of the kernel (R_1, R_2 or R_3) can be set equal to its radius of gyration if $R_j = \sqrt{5\lambda_j}$. To generate AFM image, the integrated isosurfaces of all the kernels is rasterized to a XY-grid emulating an AFM probe coming from positive Z-axis. The method is described previously in more detail (Dasgupta et al., 2020). In the current experimental data, the lateral resolution AFM images along X and Y directions are 4.545Å and 5.477Å (Uchihashi et al., 2018). To estimate the kernel surface within this area we split a pixel into 9 grids and evaluate height along z-axis based on the kernel parameters in each grid. The representative height for the pixel is taken to be maximum height over 9 grid points.

After generation of the pseudo-AFM image from a candidate model, we need to compare it against the target AFM image, which in the current study is done by structural similarity metric (SSIM) (Wang et al., 2004; Wang and Bovik, 2009), a popular 2D image comparison metric. The SSIM between two images A and B is given by,

$$SSIM(A,B) = \frac{(2\mu_A\mu_B+C_1)(2\sigma_{AB}+C_2)}{({\mu_A}^2+{\mu_B}^2+C_1)({\sigma_A}^2+{\sigma_B}^2+C_2)} \qquad (2)$$

Where, μ_A and μ_B are the average of A and B, σ_A and σ_B are the standard deviations of A and B, and σ_{AB} is the covariance of A and B. The C1 and C2 are two small constants to avoid instability, $C_1 = (K_1L)^2$ and $C_2 = (K_2L)^2$, where, L is the dynamic range of the pixel values, K_1 and K_2 are 0.01 and 0.03 as set by default in python skimage.measure.compare_ssim.

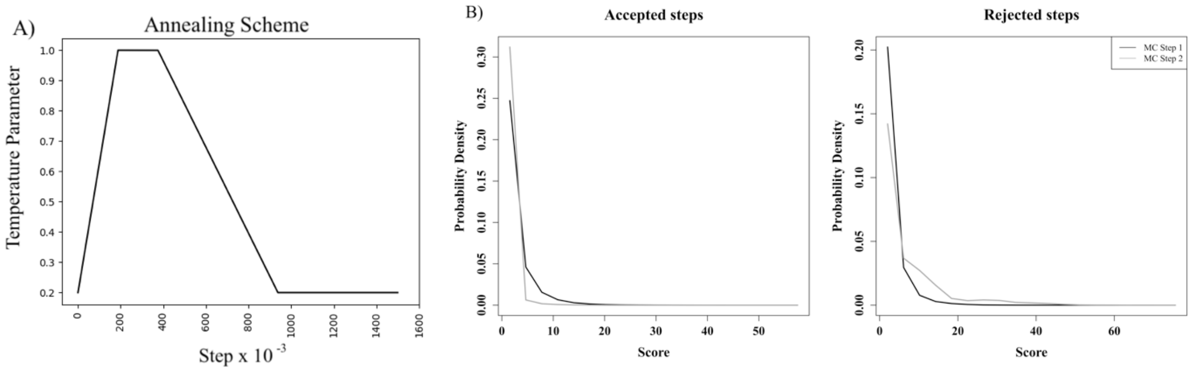

Supplementary Figure 3: A) Annealing scheme used during MC algorithm. B) Histogram of change in scores for the proposals that are accepted (left) and rejected (right) by the restraint calculation or target similarity. The two profiles are for first and second steps of MC optimization. Note that the rejected steps here are already passed in the first MC step but failed in the second step.

During the MC sampling, the temperature parameter is updated by following an annealing schedule given in Supplementary Figure 3A, where the temperature changes from 0.2 to 1.0. Here, we regard that 0.2 is the equilibrium sampling temperature, and 1.0 is a temperature where our models may undergo large changes, which are obtained by trial and error. In the MC algorithm we have two temperature parameters, but we have observed that if we scale distance scores obtained from the above SSIM (ranged between 0 and 1) by a multiplicative factor of 10000, the change in SSIM in our study is comparable to the change in restraint score in a typical move.

We have analyzed how two steps in the MC optimization process (Supplementary Figure 2) are accepting or rejecting candidate models taking one trajectory as an example. In Supplementary Figure 3B, we plot histograms of change in restraint scores (from the first MC step in our two-step optimization) and change in SSIM (from the second MC step) only when the change is positive (an upward move). Such upward moves are accepted or rejected by restraint calculation or image similarity. The majority of the proposals are rejected in the first step (around 57% of all proposals) based on restraint score and overall, around 37% of the proposals are accepted. We also observed that the proposals that are rejected in the first step are mostly due to harmonic restraint (around 78%), and the rest are due to connectivity restraint. For the accepted steps, we further analyzed that restraint scores are increasing and decreasing with nearly equal probability. Interestingly in nearly 64% of the accepted steps, the similarity to target AFM image increased (downward move in the second step).

4. Sampling restraints:

One of the key ingredients in our MC algorithm is the restraint based on 3D structure of a proposed model. To define a restraint between the kernels of Gaussian mixture model, we used correlation coefficient between a pair of kernels. The correlation coefficient between two 3D-kernels, i and j, is given by,

$$C_{ij} = \frac{O_{ij}}{\sqrt{O_{ii}O_{jj}}} \tag{3}$$

where, O_{ij} indicates overlap between kernels i and j, given by,

$$O_{ij} = \frac{1}{(2\pi)^{3/2}\left|\Sigma_{ij}\right|^{1/2}} e^{-\frac{1}{2}(\mu_i-\mu_j)^T \Sigma_{ij}{}^{-1}(\mu_i-\mu_j)} \tag{4}$$

where, $\Sigma_{ij} = \Sigma_i + \Sigma_j$. When two kernels of identical axes lengths are completely eclipsed then the correlation coefficient is 1. The restraint based on the correlation coefficient is a harmonic function, where repulsion and attraction are treated independently. For this restraint, we also need to define an equilibrium value between two kernels, $C_{ij,0}$ which we call native value. If at an instance, the correlation coefficient C_{ij} is greater than $C_{ij,0}$, then kernels are closing in, which we try to avoid due to repulsion, and if at an instance the correlation coefficient C_{ij} is smaller than $C_{ij,0}$, then kernels are going farther apart, which we try to avoid using attraction. A simplified harmonic score to those instances for M number of kernel pairs that treats attraction and repulsion in similar way can be written as,

$$E = \sum_{i,j}^{M} E_{ij} = \sum_{i,j}^{M} 0.5K_{ij}\left(C_{ij} - C_{ij,0}\right)^2 \quad (5)$$

where K_{ij} is corresponding force-constant. However, in our case we model attraction and repulsion differently. So, in the current study we define our harmonic score as follows,

$$E_{ij} = \begin{matrix} 0.5K_{ij}^{a}\left(C_{ij} - C_{ij,0}\right)^2, & if\ C_{ij} < C_{ij,0} \\ 0.5K_{ij}^{r}\left(C_{ij} - C_{ij,0}\right)^2, & if\ C_{ij} > C_{ij,0} \end{matrix} \quad (6)$$

where, K_{ij}^{a} is force-constant related to attraction and K_{ij}^{r} is force-constant related to repulsion. Next, we parameterize the force-constants in the following way. We define a relative absolute error in C_{ij} from $C_{ij,0}$ as,

$$\delta_{ij} = \left|\frac{C_{ij} - C_{ij,0}}{C_{ij,0}}\right| \quad (7a)$$

which can also be written in the form,

$$(C_{ij} - C_{ij,0})^2 = \delta_{ij}^2 C_{ij,0}^2 \quad (7b)$$

and then by combining equation (6), takes the form

$$E_{ij} = \begin{matrix} 0.5K_{ij}^{a}\delta_{ij,a}^2 C_{ij,0}^2, & if\ C_{ij} < C_{ij,0} \\ 0.5K_{ij}^{r}\delta_{ij,r}^2 C_{ij,0}^2, & if\ C_{ij} > C_{ij,0} \end{matrix} \quad (8)$$

where, $\delta_{ij,a}$ is relative error related to attraction and $\delta_{ij,r}$ is relative error related to repulsion. Next, we assume the presence of a threshold value of E_{ij} beyond which error changes drastically so that those instances are less probable. The threshold E_{ij} is given by $E_{ij,c}$ and we arbitrarily assign it a Boltzmann probability of 0.2, so for the case of repulsion we get,

$$E_{ij,c} = -KT \ln 0.2 = 0.5K_{ij}^{r}\delta_{ij,r,c}^2 C_{ij,0}^2 \quad (9a)$$

$$\text{Or, } K_{ij}^{r} = -KT \ln 0.2 \,/\, 0.5\delta_{ij,r,c}^2 {C_{ij,0}}^2 \quad (9b)$$

where, $\delta_{ij,r,c}$ is the relative error at the threshold repulsion score (relative threshold error), which is parameterizing K_{ij}^{r}. Similarly, we define $\delta_{ij,a,c}$ is the relative error at the threshold attraction score, which

is parameterizing K_{ij}^{a}. Before starting our sampling, we compute the force-constant from user-defined values of $\delta_{ij,r,c}$ and $\delta_{ij,a,c}$, and then score at any instance is calculated by equation (6) during sampling.

$\delta_{ij,r,c}$ and $\delta_{ij,a,c}$ for different types of kernel pairs are defined as follows. Here, we are mostly concerned with how kernels from neighboring chains interact with each other and how kernels within a monomer interact. Therefore, we broadly classify kernel pairs in the a) intrachain kernel pairs, b) interchain kernel pairs. Here, intrachain kernel pairs are among head, body, and tail kernels within a monomer. For the intrachain kernel pairs, the relative threshold error was set to be small so that three domains within a chain remain connected. However, with threshold errors smaller than used in the current study, the monomers become more rigid such that relative orientations between head and body kernels are fixed. We used a relative threshold error for neighboring body kernels three times higher, i.e., weaker, than for intrachain pairs. When larger values were tested, random overlaps between distant kernels occurred and the overall hexametric structure did not remain intact, such that the pore in the middle vanishes. The head kernels that are placed on the coiled-coil domain of the chains should be least restricted as they are highly flexible, and thus the head kernels between neighboring chains interact weakly. For interchain kernel pairs at the seam, we assigned a large threshold error for the attractive interactions to allow the opening/closing conformational transitions that are evident from the experimental data. Such parameter tuning was performed mostly by using AFM image 7 as it clearly shows a seam (Supplementary Table 4). For half-spiral cases, we assumed another seam (between chains C and D) opposite to the original seam between chains A and F. For these cases, we use identical parameters between C and D to that between A and F.

The native correlation coefficient, $C_{ij,0}$, is defined as follows. The bacterial ClpB structure in the AFM experimental condition is undergoing a large conformational transition (Uchihashi et al., 2018) and many AFM images were annotated as spiral and closed. For other organisms and different experimental conditions, atomically detailed symmetric closed-like structure and asymmetric spiral structure (Yokom et al., 2016; Deville et al., 2017) have been determined. Those two atomic structures are the two extreme conformations known so far with and without symmetry and we assumed that the conformations observed by AFM experiments are within the conformational space between these two extreme conformations. Therefore, we prepare two low-resolution initial models based on Gaussian mixture model based on those two extreme cases and the average correlation coefficient between equivalent kernel pairs is used as native correlation coefficients.

We also used a restraint score based on overlapping region between a pair of kernels to define a connected region. This restraint is used for the kernel pairs within a monomer and is used to keep the overall shape of the monomer and to ensure that two kernels do not slip along each other. Such connections between the kernels are shown in Fig. 2I where the head and body kernels are connected in a monomer near the AAA1+ domain part of the body kernel, and the body and tail kernels are connected near the AAA2+ part of the body kernel. For the connectivity restraint we initially define a pair of phantom particles (p_i and p_j) each on top of the other in the overlapping region of a kernel pair i,j (Supplementary Figure 5) affixed to the respective (initially the distance between p_i and p_j, d_{ij} = 0). During the random move of one of the kernels the affixed phantom particle moves with the kernel, therefore the phantom particles are separated. We added a condition that the distance between these two phantom particles d_{ij} < 5Å and always p_i and p_j remain in the overlapping region. Otherwise, such a move is rejected in the first step of MC.

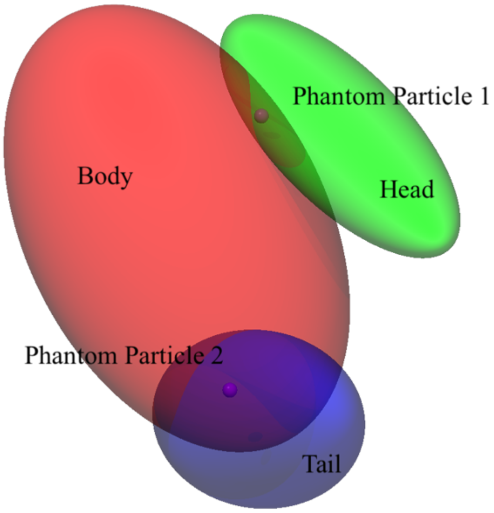

Supplementary Figure 4: Initial positions of phantom particles for connectivity restraint within a monomer between body and head kernels and between body and tail kernels.

Supplementary Table 4: Parameters for harmonic restraint score

Kernel pair type	Relative threshold error for repulsion ($\delta_{ij,r,c}$)	Relative threshold error for attraction ($\delta_{ij,a,c}$)
Intrachain	0.05	0.05
Interchain neighboring body kernels#	0.15	0.15
Interchain neighboring head kernels#	0.50	0.50
Interchain neighboring tail kernels#	0.15	0.50
Interchain neighboring kernels in general	0.25	9.99$
Interchain neighboring kernels at the seam	0.15	9.99$

#Except for the neighboring kernels at the seam

$9.99 signifies a large value for the relative threshold error, effectively any change in attraction do not add significant contribution to the score.

Supplementary Table 5: Clustering of selected candidate models at the end of sampling that started from R and S initial models. The method for clustering is discussed in the main text. For each type, in the left the clustering is shown in 2-dimentional projected space along principal components axes, and the converged representative model is shown as a cyan triangle point and the cluster medians are shown as black circle. In the right the cluster sizes are shown in a pie-chart. The cluster that includes converged representative model is exploded. The cluster names are given in the legend in the left and annotated in the pie-chart.

AFM Image index	Initial model type	Clusters along principal directions	Cluster Size
1	R	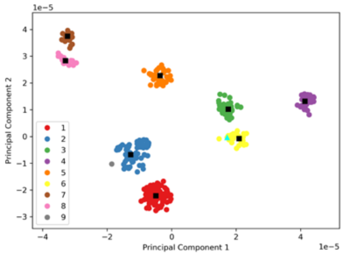	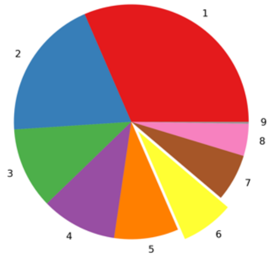
	S	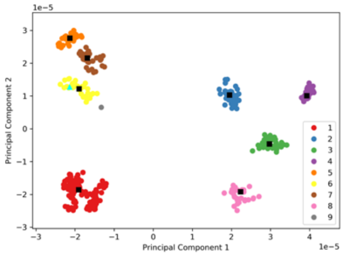	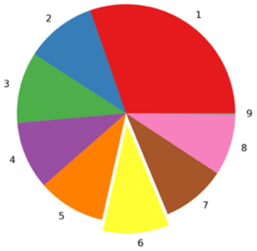
2	R	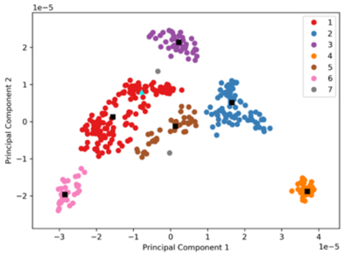	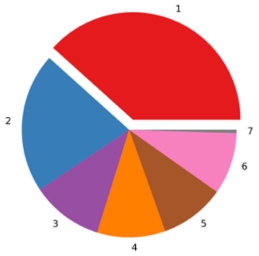
	S	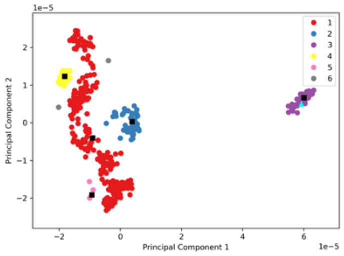	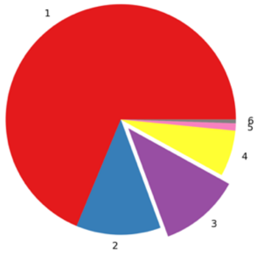

3	R	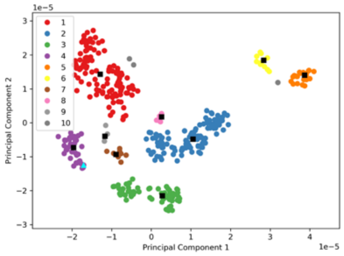	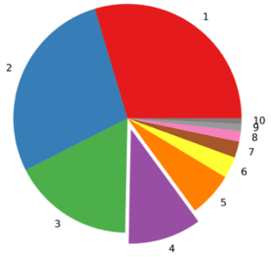
	S	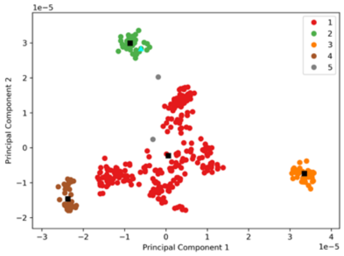	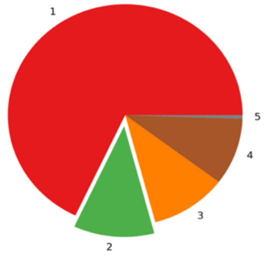
4	R	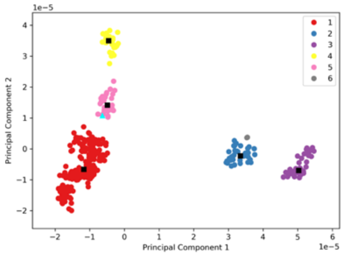	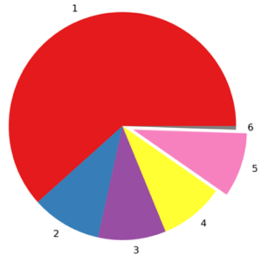
	S	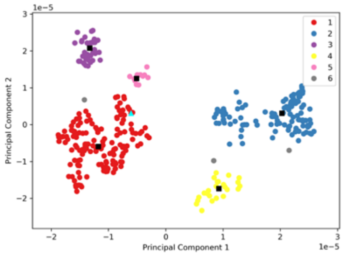	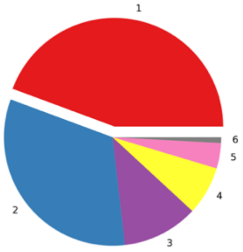
5	R	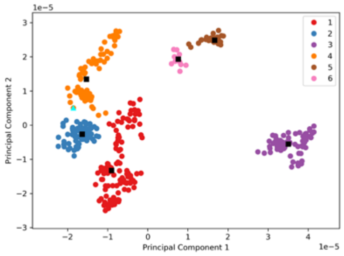	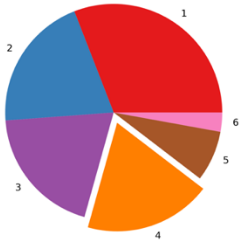
	S	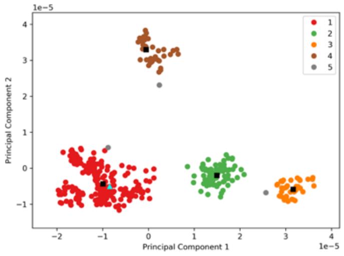	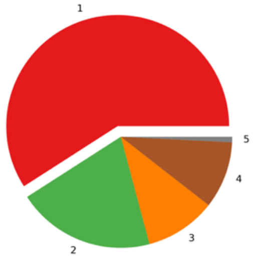

6	R	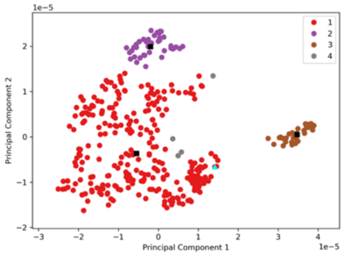	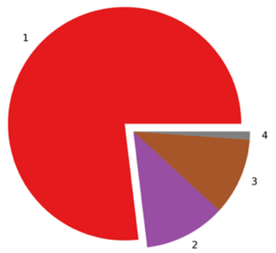
	S	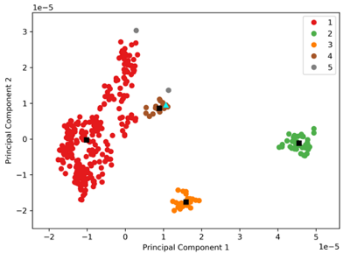	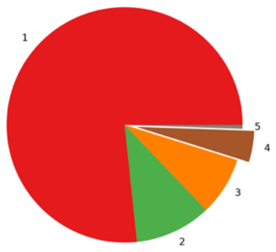
7	R	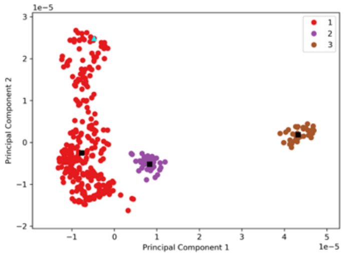	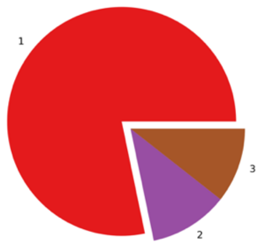
	S	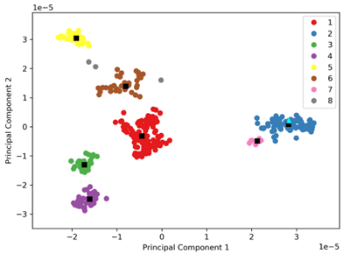	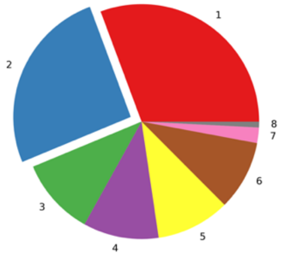
8	R	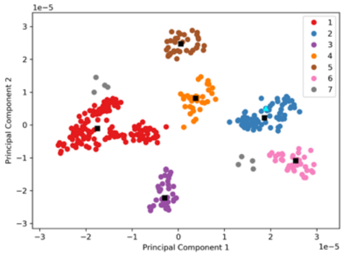	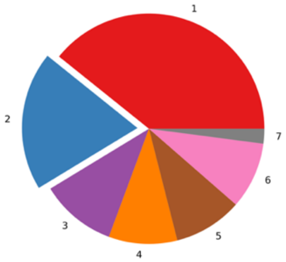
	S	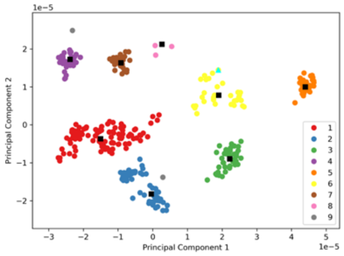	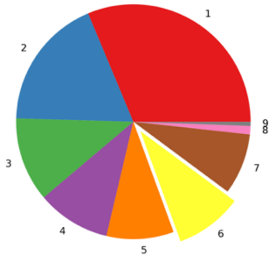

9

R

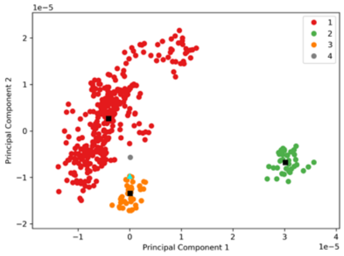

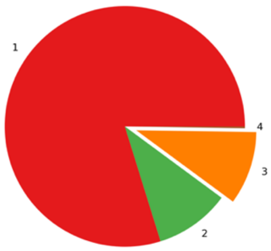

S

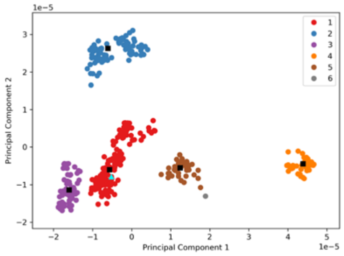

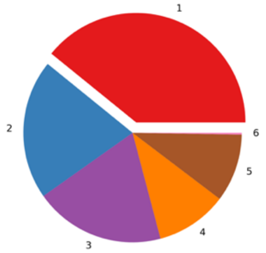

10

R

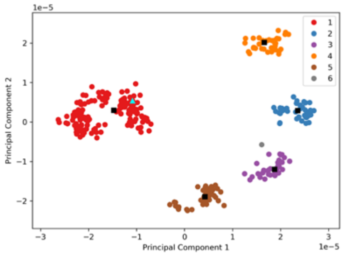

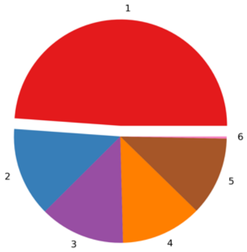

S

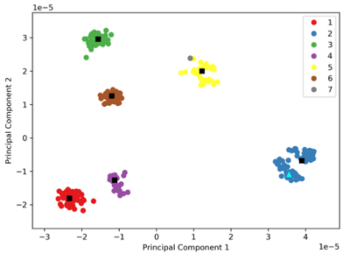

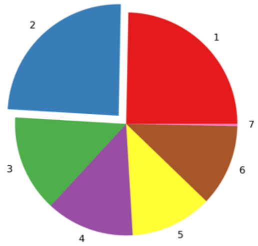

11

R

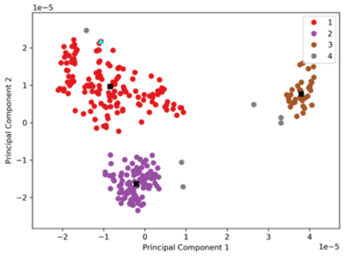

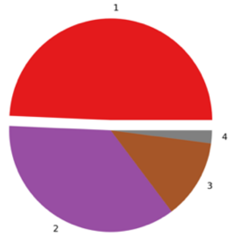

S

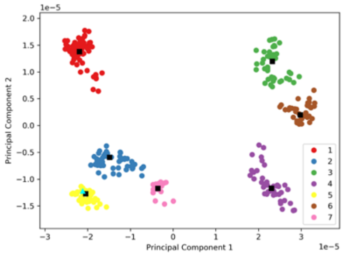

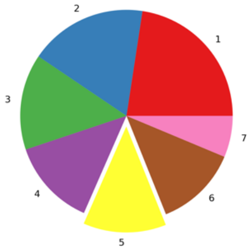

12	R	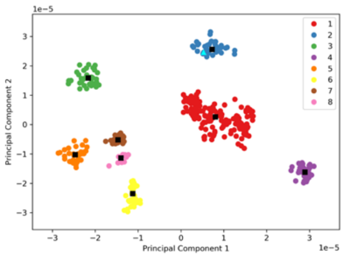	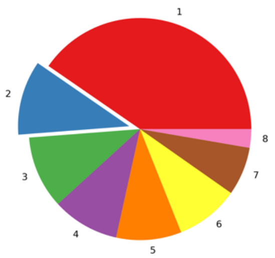
	S	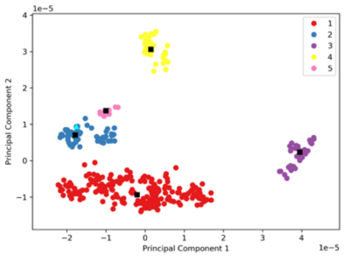	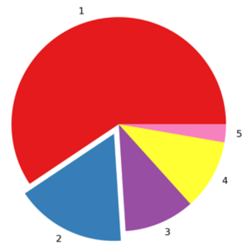

A)

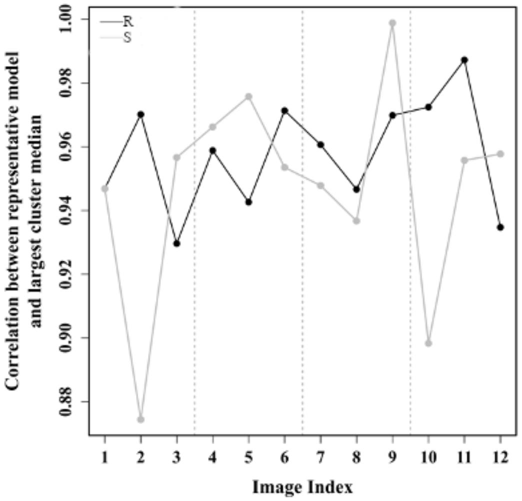

B)

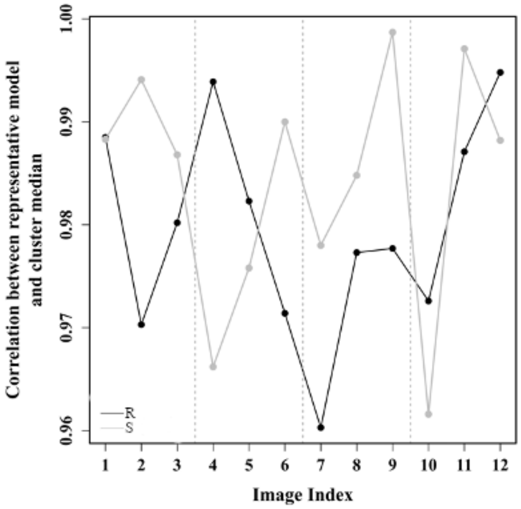

Supplementary Figure 5. Comparison of converged representative model for each AFM image derived from R or S models to the respective cluster medians. In A) comparison to the largest cluster median, and in B) comparison to the cluster median to which representative model belongs. In A) for most of the cases the similarity by $CC3D_{density} > 0.9$, except of AFM image 10 (half-spiral) and AFM image 2 (open) from S. Visual inspection confirms such differences are due to orientation of flexible coiled-coil domain or C-terminal domain, which is only partially observed or not observed in AFM images.

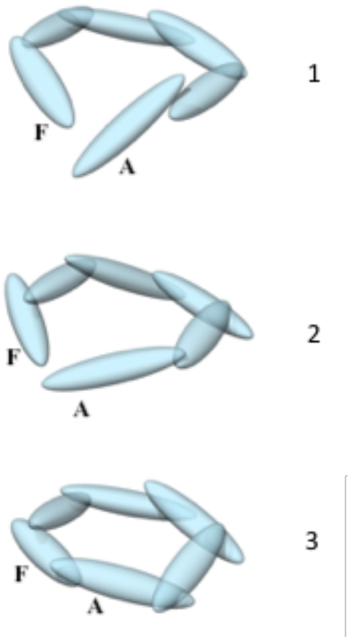

Supplementary Figure 6: Orientations of head kernels in converged models from R (Fig. 4, main text, leftmost column, rotated here around x-axis by 45º). The annotations ‘1’, ‘2’ and ‘3’ are corresponding AFM images. ‘A’ and ‘F’ are chains from which head kernels originated. In AFM image 1, both the head kernels from A and F are facing downward, while in AFM image 3, a clear hexameric arrangement is seen.

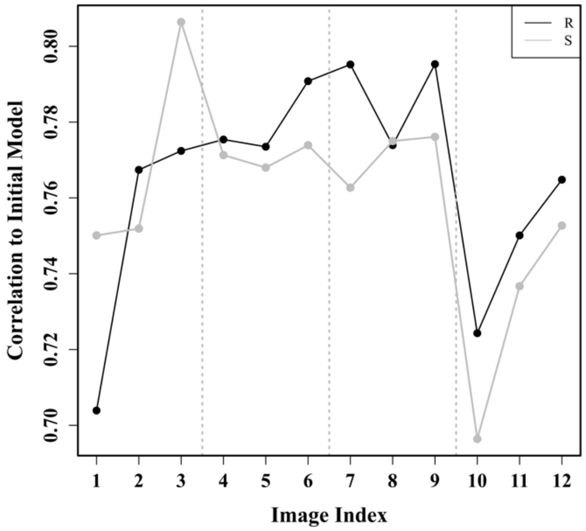

Supplementary Figure 7: Correlation coefficient ($CC3D_{density}$) between the converged models (final candidate solutions) for each image and corresponding initial model R and S. The horizontal axis includes AFM image indices 1 to 12 and in vertical axis the $CC3D_{density}$ are shown for R and S separately. The vertical dotted-line separates different classes of AFM images.

Supplementary Table 6: The superposition of converged representative models for each AFM image derived from R or S (in dark red, mesh style) and respected largest cluster median (in light green, surface style).

AFM Image Index	From R initial model	From S initial model
1	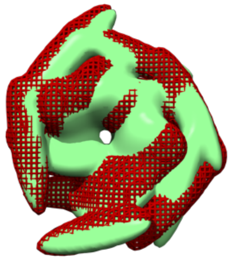	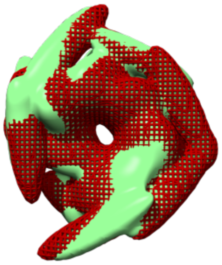
2	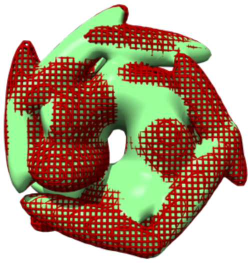	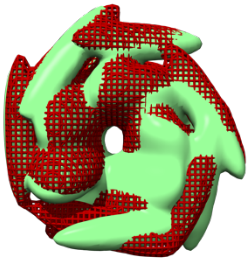
3	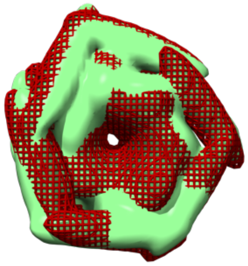	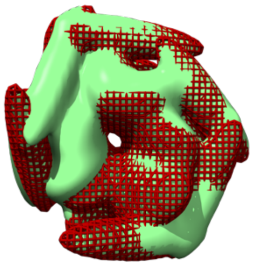
4	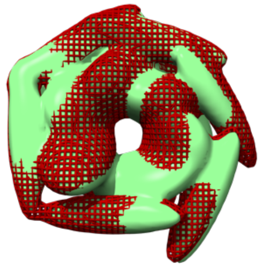	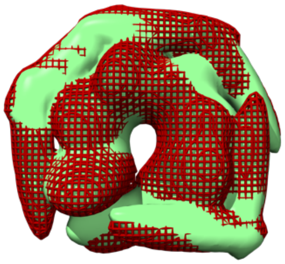

5	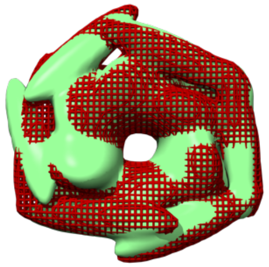	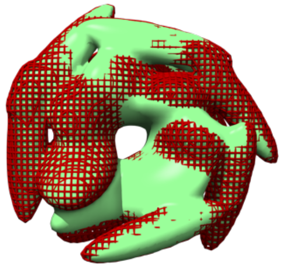
6	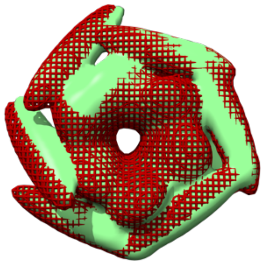	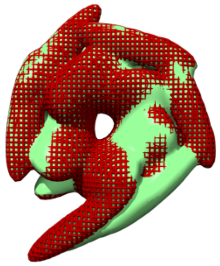
7	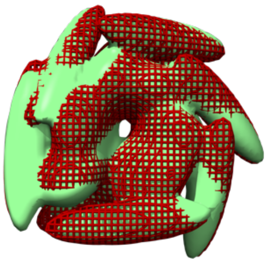	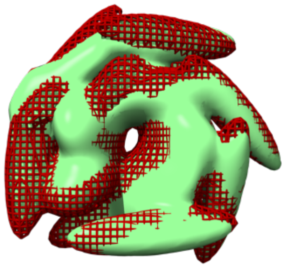
8	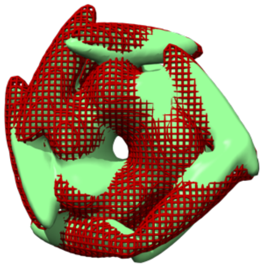	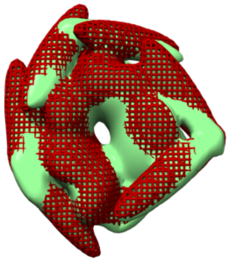
9	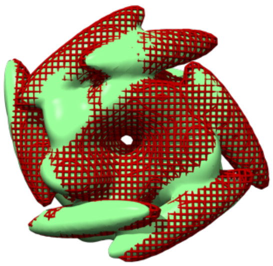	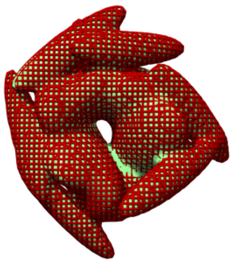

10	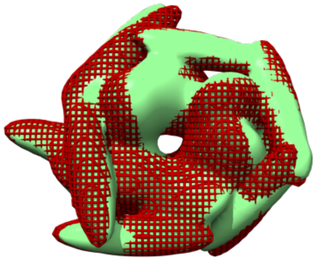	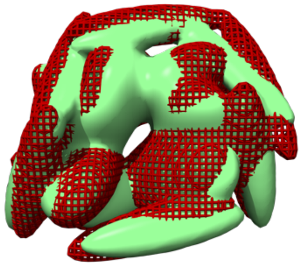
11	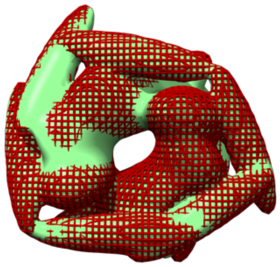	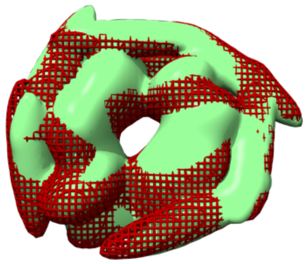
12	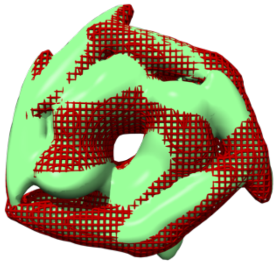	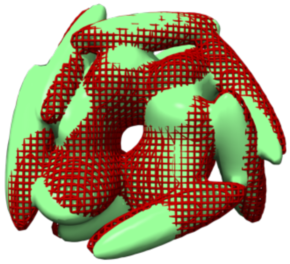

References:

Dasgupta, B., Miyashita, O., and Tama, F. (2020). Reconstruction of low-resolution molecular structures from simulated atomic force microscopy images. *Biochim. Biophys. Acta Gen. Subj.* 1864, 129420. doi:10.1016/j.bbagen.2019.129420.

Deville, C., Carroni, M., Franke, K. B., Topf, M., Bukau, B., Mogk, A., et al. (2017). Structural pathway of regulated substrate transfer and threading through an Hsp100 disaggregase. *Sci. Adv.* 3, e1701726. doi:10.1126/sciadv.1701726.

Kawabata, T. (2008). Multiple subunit fitting into a low-resolution density map of a macromolecular complex using a Gaussian mixture model. *Biophys. J.* 95, 4643–4658. doi:10.1529/biophysj.108.137125.

Lee, S., Sowa, M. E., Watanabe, Y., Sigler, P. B., Chiu, W., Yoshida, M., et al. (2003). The structure of ClpB: a molecular chaperone that rescues proteins from an aggregated state. *Cell* 115, 229–240.

Madeira, F., Park, Y. mi, Lee, J., Buso, N., Gur, T., Madhusoodanan, N., et al. (2019). The EMBL-EBI search and sequence analysis tools APIs in 2019. *Nucleic Acids Research* 47, W636–W641. doi:10.1093/nar/gkz268.

Meng, E. C., Pettersen, E. F., Couch, G. S., Huang, C. C., and Ferrin, T. E. (2006). Tools for integrated sequence-structure analysis with UCSF Chimera. *BMC Bioinformatics* 7. doi:10.1186/1471-2105-7-339.

Pettersen, E. F., Goddard, T. D., Huang, C. C., Couch, G. S., Greenblatt, D. M., Meng, E. C., et al. (2004). UCSF Chimera - A visualization system for exploratory research and analysis. *J. Comput. Chem.* 25, 1605–12. doi:10.1002/jcc.20084.

Schneider, C. A., Rasband, W. S., and Eliceiri, K. W. (2012). NIH Image to ImageJ: 25 years of image analysis. *Nat. Methods* 9, 671–675. doi:10.1038/nmeth.2089.

Uchihashi, T., Watanabe, Y.-H., Nakazaki, Y., Yamasaki, T., Watanabe, H., Maruno, T., et al. (2018). Dynamic structural states of ClpB involved in its disaggregation function. *Nat. Commun.* 9, 2147. doi:10.1038/s41467-018-04587-w.

Wang, Z., and Bovik, A. C. (2009). Mean squared error: Love it or leave it? A new look at Signal Fidelity Measures. *IEEE Signal Processing Magazine* 26, 98–117. doi:10.1109/MSP.2008.930649.

Wang, Z., Bovik, A. C., Sheikh, H. R., and Simoncelli, E. P. (2004). Image quality assessment: from error visibility to structural similarity. *IEEE Trans. Image Process.* 13, 600–612. doi:10.1109/tip.2003.819861.

Webb, B., and Sali, A. (2014). Comparative Protein Structure Modeling Using MODELLER. *Curr. Protoc. Bioinformatics* 47, 5.6.1-5.6.32. doi:10.1002/0471250953.bi0506s47.

Yokom, A. L., Gates, S. N., Jackrel, M. E., Mack, K. L., Su, M., Shorter, J., et al. (2016). Spiral architecture of the Hsp104 disaggregase reveals the basis for polypeptide translocation. *Nat. Struct. Mol. Biol.* 23, 830–837. doi:10.1038/nsmb.3277.
